# Electronic Coupling and Electrocatalysis in Redox Active Fused Iron Corroles

**DOI:** 10.1021/acs.inorgchem.2c01389

**Published:** 2022-12-13

**Authors:** Amir Mizrahi, Susovan Bhowmik, Arun K. Manna, Woormileela Sinha, Amit Kumar, Magal Saphier, Atif Mahammed, Moumita Patra, Natalia Fridman, Israel Zilbermann, Leeor Kronik, Zeev Gross

**Affiliations:** † Chemistry Department, Nuclear Research Centre Negev, Beer-Sheva84190, Israel; ‡ Schulich Faculty of Chemistry, Technion Institute of Technology, Haifa3200003, Israel; § Bankura Sammilani College (W.B), Bankura722102, India; ∥ Department of Molecular Chemistry and Materials Science, Weizmann Institute of Science, Rehovot76100, Israel; ⊥ Department of Chemistry, BITS PilaniK K Birla Goa Campus, NH17B, Zuarinagar, Goa403726, India; # Kazi Nazrul University (W.B), Asansol713340, India; ∇ Chemistry Department, Ben-Gurion University of the Negev, Beer-Sheva84105, Israel

## Abstract

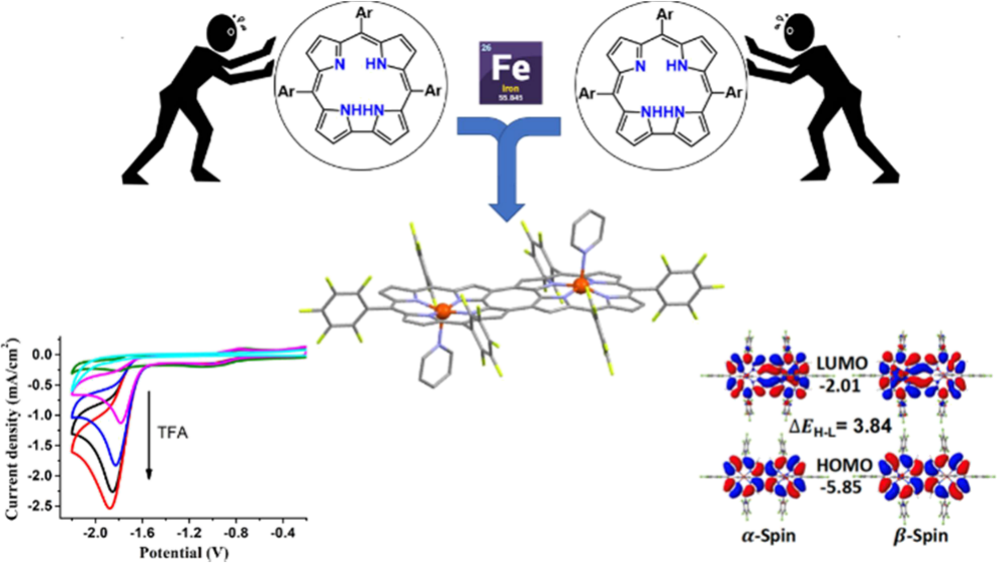

Conjugated arrays composed of corrole macrocycles are increasingly more common, but their chemistry still lags behind that of their porphyrin counterparts. Here, we report on the insertion of iron(III) into a β,β-fused corrole dimer and on the electronic effects that this redox active metal center has on the already rich coordination chemistry of [H_3_tpfc] COT, where COT = cyclo-octatetraene and tpfc = tris(pentafluorophenyl)corrole. Synthetic manipulations were performed for the isolation and full characterization of both the 5-coordinate [Fe^III^tpfc(py)]_2_COT and 6-coordinate [Fe^III^tpfc(py)_2_]_2_COT, with one and two axial pyridine ligands per metal, respectively. X-Ray crystallography reveals a dome-shaped structure for [Fe^III^tpfc(py)]_2_COT and a perfectly planar geometry which (surprisingly at first) is also characterized by shorter Fe–N (corrole) and Fe–N (pyridine) distances. Computational investigations clarify that the structural phenomena are due to a change in the iron(III) spin state from intermediate (*S* = 3/2) to low (*S* = 1/2), and that both the 5- and 6-coordinated complexes are enthalpically favored. Yet, in contrast to iron(III) porphyrins, the formation enthalpy for the coordination of the first pyridine to Fe(III) corrole is more negative than that of the second pyridine coordination. Possible interactions between the two corrole subunits and the chelated iron ions were examined by UV–Vis spectroscopy, electrochemical techniques, and density functional theory (DFT). The large differences in the electronic spectra of the dimer relative to the monomer are concluded to be due to a reduced electronic gap, owing to the extensive electron delocalization through the fusing bridge. A cathodic sweep for the dimer discloses two redox processes, separated by 230 mV. The DFT self-consistent charge density for the neutral and cationic states (1- and 2-electron oxidized) reveals that the holes are localized on the macrocycle. A different picture emerges from the reduction process, where both the electrochemistry and the calculated charge density point toward two consecutive electron transfers with similar energetics, indicative of very weak electron communication between the two redox active iron(III) sites. The binuclear complex was determined to be a much better catalyst for the electrochemical hydrogen evolution reaction (HER) than the analogous mononuclear corrole.

## Abstract 2

Investigation of the bis-iron chelated β,β-fused corrole dimer uncovered different communications between the metal centers as a function of oxidation and coordination states, as well as improved performance relative to the monometallic analogue regarding the electrochemical catalysis of the HER.

## 1 Introduction

**Figure 1:**
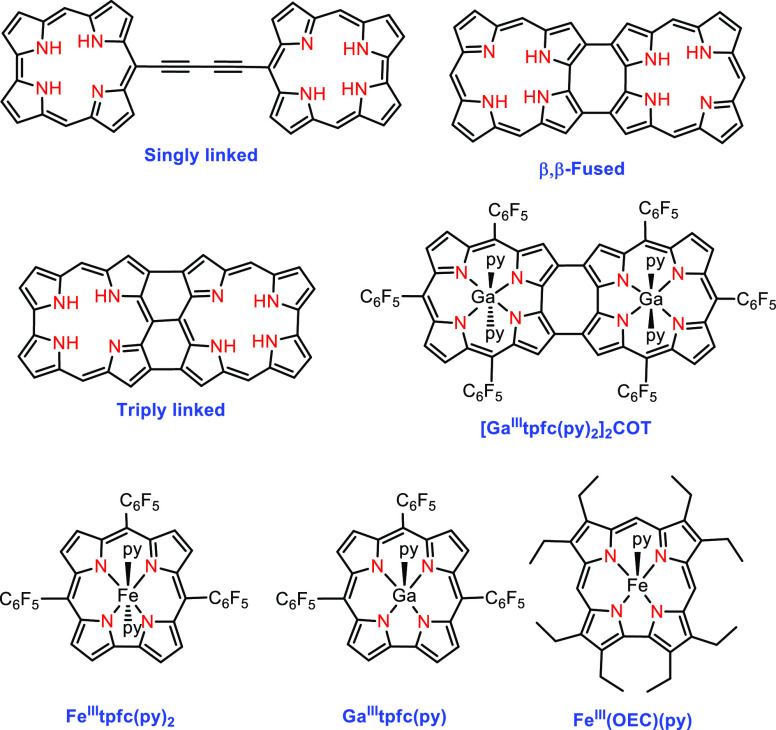
Substituent-omitted structures of singly, doubly (=fused), and triply linked corroles and the chemical structures of the β,β-fused gallium corrole dimer ([Ga^III^tpfc(py)_2_]_2_COT) and of the monomeric gallium and iron (Fe^III^tpfc(py)_2_ and Fe^III^(OEC)(py)) corroles. This study focuses on the iron complexes of the β,β-fused corrole dimer in which all *meso*-C positions are substituted by C_6_F_5_ rings, abbreviated as [Fetpfc(*L*)_*n*_]_2_COT with *n* = 1 or 2.

Conjugated arrays based on porphyrins, and to a lesser extent corroles, have been receiving much attention owing to their attractive electronic,^1−3^ optical,^4−6^ and electrochemical^7,8^ properties. One area of focus is the development of synthetic strategies for various modes of conjugation, which may be classified by the number of C atoms involved in each of the chromophores: bridging by π-conducting moieties that link^9,10^ one *meso*-C atom;^11^ 8-membered ring formation by using two β-pyrrole C atoms;^11−13^ heavily fused derivatives in which 4 C atoms are involved (Figure 1).^14,5^ Some of the present authors have previously contributed a specific example of the second above-mentioned kind, wherein two corroles are bridged by what was formally a cyclo-octatetraene (COT) subunit (Figure 1, β,β-fused structure).^15^ This was initially pursued to determine if this formally antiaromatic moiety is conducting or isolating. By way of resolving that the former case is in effect, multiple interesting phenomena regarding the communication between the corrole subunits upon oxidation and reduction have also been determined.

The above investigations, performed on chelates with the non redox-active gallium(III) ion (Figure 1, [Ga^III^tpfc(py)_2_]_2_COT), paved the way for addressing more interesting and intellectually demanding cases, notably iron complexes of the same dimer. The motivation was to comprehend if and how strongly the two redox active metal centers interact with each other and, based on that, to decide if we could advance these compounds to be of use for energy relevant catalytic processes.

We have now addressed this challenge by preparing and characterizing bis-iron bis-corrole complexes. The monomeric analogue has been isolated as bis-ether iron(III), bis-pyridine iron(III) (Figure 1, Fe^III^(tpfc)(py)_2_), and iron(IV) chloride (*formal oxidation state*)^16^ corrole, while a monopyridine iron(III) complex was reported with octaethylcorrole (OEC) (Figure 1, Fe^III^(OEC)(py)).^17,18^ The magnetism changes very much in this series: the bis-ether and monopyridine complexes are intermediate spin (*S* = 3/2), the bis-pyridine is low spin (*S* = 1/2), and the neutral iron chloride corrole is *S* = 1 due to a formal Fe(IV) oxidation state and some contribution from a Fe(III) corrole cation radical formulation. We have now prepared the iron complexes of the fused corrole dimer at different coordination states: both the 5- and 6-coordinated iron(III) complexes [Fe^III^tpfc(Py)]_2_COT and [Fe^III^tpfc(py)_2_]_2_COT, respectively, where tpfc = tris(pentafluorophenyl)corrole and py = pyridine. The complexes were characterized by a blend of experimental and theoretical methodologies, and also applied as catalysts for the hydrogen evolution reaction as to explore possible benefits of the bimetallic complexes.

## 2 Experimental and Computational Details

### 2.1 Materials

All the routine chemical reagents and solvents were purchased from commercial sources and were purified by standard procedures before use. The free-base COT-bridged corrole dimer (H_3_tpfc)_2_COT was synthesized according to an earlier reported procedure.^19^

### 2.2 Synthesis

#### 2.2.1 [Fe^III^tpfc(py)]_2_COT with One Pyridine Ligand for Each Iron Center

The free-base corrole dimer (80 mg, 0.05 mmol) was dissolved in 30 mL dry tetrahydrofuran (THF) under N_2_ followed by the addition of FeCl_2_ (0.25 g, 2 mmol). The mixture was heated to reflux, and the process was followed by TLC (silica, *n*-hexane/CH_2_Cl_2_ 2:1). After completing the iron insertion, the reaction mixture was cooled to 25 °C, and the solvent was evaporated. The resulting solid material was dissolved in THF and purified by chromatography over a short column (10 cm long, 2 cm diameter, with silica gel and THF as an eluent). [Fe^III^tpfc(py)]_2_COT was obtained by recrystallization with a drop of pyridine from an aerobic solution of cyclohexane and benzene, in 53% yield (49 mg, 0.0265 mmol). ^1^H nuclear magnetic resonance (NMR) (400 MHz, toluene-*d*_8_) δ = 73.00 (axial pyridine ligand), −3.56 (β-pyrr-H), −54.49 (β-pyrr-H), −131.40 (β-pyrr-H) ppm. ^19^F NMR (377 MHz, pyridine-*d*5) δ = −112.43 (*ortho*-F), −119.78 (*ortho*-F), −149.00 (*para*-F), −150.05 (*para*-F), −156.37 (*meta*-F), −157.33 (*meta*-F). UV/Vis (CH_2_Cl_2_): λ_max_ (ε, M^–1^ cm^–1^): 382 (17,400), 433 (15,500), 638 (6300), 722 (7400), 821(3300) nm. MS^+^ (turn-over frequency, positive mode) for: C_74_H_12_F_30_Fe_2_N_8_ [M-2py]: *m*/*z* = 1693.940 (calcd), 1693.975 (observed); C_79_H_17_F_30_Fe_2_N_9_ [M-py]: *m*/*z* = 1772.983 (calcd), 1772.936 (observed).

#### 2.2.2 [Fe^III^tpfc(py)_2_]_2_COT with Two Pyridine Axial Ligands for Each Iron Center

[Fe^III^tpfc(py)_2_]_2_COT was obtained by dissolving [Fe^III^tpfc(py)]_2_COT in pure pyridine and the subsequent evaporation of the solvent (85% yield from Fe^III^tpfc(py)]_2_COT). ^1^H NMR (400 MHz, pyridine-*d*_5_) δ = 0.00 (β-pyrr-H), −38.59 (β-pyrr-H), −114.95 (β-pyrr-H) ppm. ^19^F NMR (377 MHz, pyridine-*d*5) δ = −121.98 (*ortho*-F, 4F), −133.17 (*ortho*-F, 2F), −153.02 (t, *J* = 22 Hz, *para*-F, 1F), −153.85 (t, *J* = 22 Hz, *para*-F, 2F), −160.98 (*meta*-F, 2F), −161.41 (*meta*-F, 4F). UV/Vis (pyridine): λ_max_ (ε, M^–1^ cm^–1^): 389 (13,700), 434 (14,100), 640 (6340), 685 (7000), 710 (6460), 800 (3100) nm. MS is identical to that of Fe^III^tpfc(py)]_2_COT due to losing of the pyridine axial ligands.

### 2.3 Computation

All the density functional theory (DFT) calculations presented in this work were performed using version 4.3 of Q-Chem.^20^ Geometry optimization was performed in the gas-phase, while considering different possible spin states, using ωB97X-D.^21^ This is a range-separated hybrid functional with 100% long-range Fock exchange, which also accounts for dispersive interactions, and has been previously recommended for the determination of geometry and spin-state energetics in Fe–porphyrin complexes.^22^ Normal modes were calculated at each optimized geometry to confirm that the minimum energy structure has been reached. The 6-311G(d,p) basis set was used for all nonmetallic elements (H, C, N, and F) and the 6-31G(d,p) basis set was used for Fe. Redox (electron addition and removal) energies were computed using optimal tuning^23^ of the range-separated hybrid functional LRC-*ω*PBEh,^24^ previously found to be useful for metal–organic complexes,^25−27^ using the 6-31G(d,p) basis set for all elements. Optical properties were calculated using the same functional and basis set within linear-response time-dependent DFT.^23^

## 3 Results and Discussion

Dark green X-ray quality crystals of the 5-coordinate [Fe^III^tpfc(py)]_2_COT were obtained by the slow diffusion of n-hexane into a dichloromethane solution in the presence of pyridine. The dissolution of [Fe^III^tpfc(py)]_2_COT in pure pyridine and slow evaporation led to the isolation of 6-coordinate [Fe^III^tpfc(py)_2_]_2_COT crystals, suitable for X-ray analysis.

**Figure 2:**
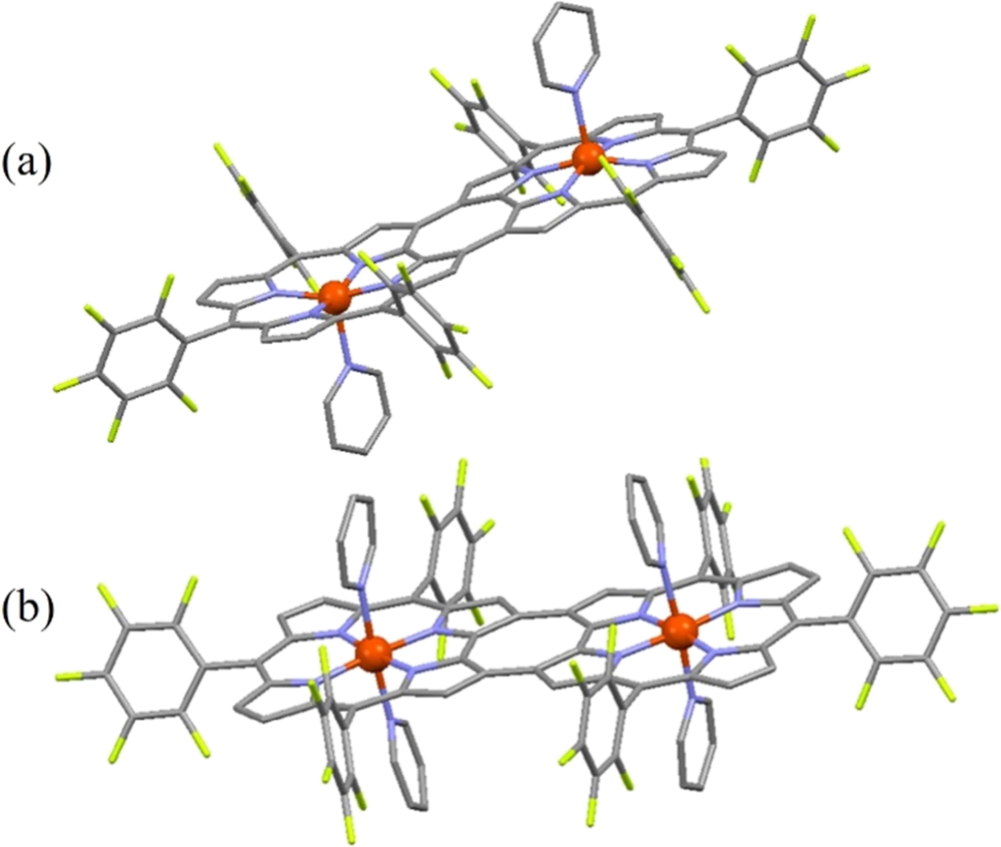
Crystal structures of [Fe^III^tpfc(py)]_2_COT (a) and [Fe^III^tpfc(py)_2_]_2_COT (b).

X-ray diffraction analysis of [Fe^III^tpfc(py)]_2_COT revealed that each subunit displays a 5-coordinate domed complex, wherein the Fe(III) ion is 0.39 Å above the corrole C_19_N_4_ plane and directed toward the axial pyridine (Figure 2a). The relative orientation of the axial pyridines on each metal center is *anti*, similar to what has been observed for the gallium^15^ and cobalt^13^ bis-corrole complexes. As shown in Figure S11a, the DFT-optimized structure reveals a similar dome-shaped complex, with the Fe(III) being 0.27 Å above the corrole C_19_N_4_ plane, with an average distance of 2.16 Å between the pyridine axial ligand and the Fe(III) center (Fe–N_py_). Furthermore, the *anti*- conformation was found to be more stable than the *syn* one by ∼1.1 kcal/mol, in agreement with the experimentally determined crystal structure. The packing diagram demonstrates weak π–π interactions [Py–Py distance of 4.064 (6) Å] between the coordinated pyridines from the neighboring dimers. A similar interaction between the benzene molecules (solvent) and coordinated pyridine [Py–C_6_H_6_ distance is 3.784 (4) Å] is notable (Figure S12).

X-ray diffraction analysis of the dark green crystals formed in pure pyridine revealed the formation of the 6-coordinate complex, [Fe^III^tpfc(py)_2_]_2_COT (Figure 2b). Each metal ion is axially coordinated by two pyridine molecules, which form identical bond lengths with it [2.019(7) Å] and are in an almost perfect coplanar arrangement. The metal ion is now essentially within the C_19_N_4_ corrole plane (only 0.0014 Å out of plane). A perfectly planar geometry has also been obtained from the gas-phase DFT calculations for the bis-py complex, with an average Fe–N_py_ distance of 2.05 Å (Figure S11b).

Comparison of the 5- and 6-coordinated iron(III) bis-corrole complexes to the previously reported monomeric iron(III) corroles, Fe^III^(OEC)(py) and Fe^III^(tpfc)(py)_2_, respectively, reveals a similar trend of shortening in the Fe–N_c_ and Fe–N_py_ bond lengths upon the coordination of a second pyridine molecule. While the shorter Fe–N_c_ bond lengths in [Fe^III^tpfc(py)_2_]_2_COT (Table S1) and in its monomeric analogue, Fe^III^tpfc(py)_2_,^15^ are expected due to the formation of an almost perfect coplanar arrangement, the shortening of Fe–N_py_ bond lengths upon the formation of [Fe^III^tpfc(py)_2_]_2_COT from [Fe^III^tpfc(py)]_2_COT may indicate a change in the Fe(III) spin state. Comparison with the monomeric analogues reveals that the 5-coordinated Fe^III^OEC(py) with a Fe–N_py_ bond length of 2.188(2) Å has an intermediate spin state (*S* = 3/2),^17^ whereas the 6-coordinated Fe^III^tpfc(py)_2_ with an average Fe–N_py_ bond length of 2.029(5) Å and 2.032 is in a low spin state (*S* = 1/2).^28^ For both cases, a difference greater than 0.1 Å was observed, as may be further appreciated by the selected crystallographic parameters reported in Table S1. Similarly reduced Fe–N_py_ distances in the planar [Fe^III^tpfc(py)_2_]_2_COT complex, relative to the dome-shaped [Fe^III^tpfc(py)]_2_COT complex, were also found computationally (Figure S11). However, a reduction in the Fe–N_c_ lengths was not observed computationally and this suggests a plausible solid-state effect. According to the calculations, the presence of bis-py in the former complex induces a stronger ligand-field. This is responsible for the change in the iron(III) spin state from *S* = 3/2 in [Fe^III^tpfc(py)]_2_COT to *S* = 1/2 in [Fe^III^tpfc(py)_2_]_2_COT.

The structural features of the COT moiety in the iron bis-corrole complexes are almost identical to those found in gallium(III) and cobalt(III) bis-corrole complexes—a planar structure and shorter bond distances for the C–C bonds that are shared by the COT moiety and the corrole subunits, compared to the two bridging bonds (Table S2). DFT-optimized structures (Figure S11) also uncover similar C–C bond distances for the bridging COT moiety in both complexes, thus strengthening the arguments of conjugation through it.

**Figure 3:**
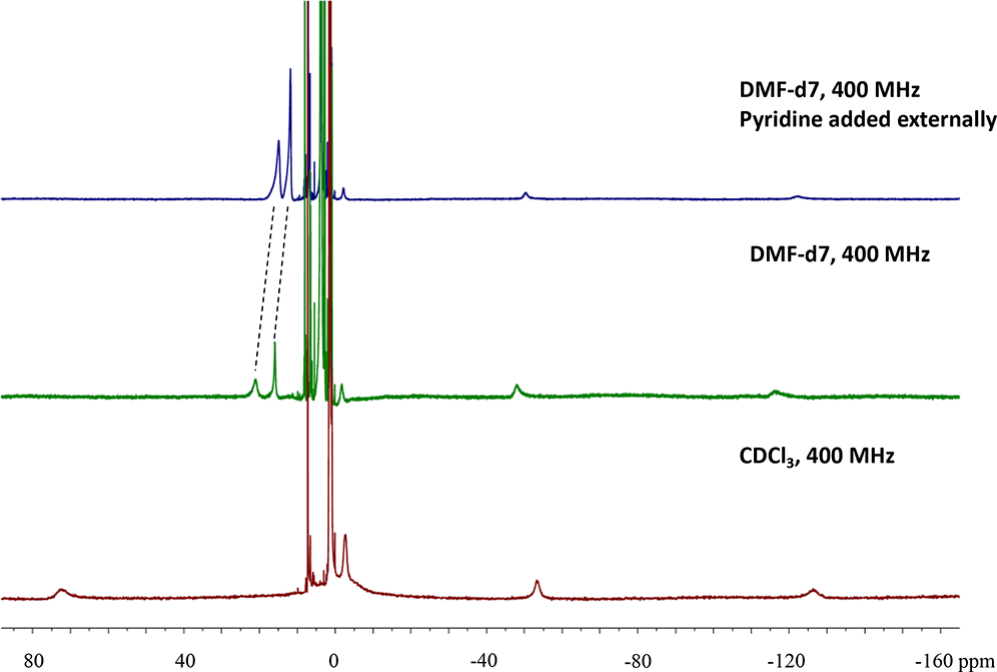
^1^H-NMR spectra of [Fe^III^tpfc(py)]_2_COT in CDCl_3_ and DMF-d_7_.

The ^19^F-NMR spectrum of the 5-coordinate iron(III) bis-corrole dimer [Fe^III^tpfc(py)]_2_COT in both CDCl_3_ and toluene (Figures S1b and S3b) displays three sets of resonances corresponding to *ortho*-F, *para*-F, and *meta*-F (from left to right, all in 2:1 ratio), similar to the ^19^F NMR spectrum of the mononuclear Fe^III^tpfc(py)_2_.^16^ For gaining information about the first coordination sphere of [Fe^III^tpfc(py)]_2_COT *in solution*, its ^1^H NMR spectrum was recorded in both noncoordinating CDCl_3_ and potentially coordinating dimethylformamide d_7_ (DMF-*d*_7_) (Figure 3). Signals that may be assigned with high confidence to β-pyrrole C–H (−126.3, −53.4, and −2.76 ppm) were hardly affected, suggesting that the solvation of [Fe^III^tpfc(py)]_2_COT does not induce any change in the spin state. The +72.3 ppm signal, corresponding to the pyridine that is directly bound to the paramagnetic center,^28,29^ is clearly seen in CDCl_3_, whereas it is located at +17 ppm in DMF-*d*_7_ and shifts to a higher field upon the addition of pyridine. This is fully consistent with the fraction of pyridine-coordinated iron(III) being larger in the noncoordinating solvent. To quantify the pyridine ligand binding strength in the two complexes, we calculated the formation enthalpy (ΔH) for the 5- (eqs 1 and 2) and 6 (eqs 3 and 4)-coordinated complexes as follows:
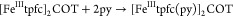
1
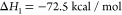
2
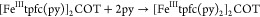
3
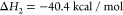
4

This reveals that the formation of both complexes is enthalpically favored, relative to the 4-coordinated complex. This is fundamentally different from iron(III) porphyrins, for which the second process is much more favored than the first.^30,31^

The ^1^H NMR features of the bis-iron(III) corrole were further examined at various temperatures, by dissolving [Fe^III^tpfc(py)]_2_COT in two solvents: toluene-*d*_8_, where it remains 5-coordinate and pyridine-*d*_5_, wherein it becomes 6-coordinate. All β-pyrrole protons signals shifted to a progressively higher field as the temperature was decreased in the former case, but in pyridine-*d*_5_ one of the three β-pyrrole resonances shifted to low-fields. While the Curie plots (β-pyrrole chemical shifts against inverse of the temperature, Figure S4) were straight in both solvents, consistent with no changes in the spin state in the examined temperature range, the slope was much larger in toluene than in pyridine. Both phenomena, as well as much the smaller width at half maxima of both the ^1^H and ^19^F resonances in pyridine, are consistent with *S* = 3/2 and *S* = 1/2 spin states on each iron center of the 5-coordinate [Fe^III^tpfc(py)]_2_COT and 6-coordinate [Fe^III^tpfc(py)_2_]_2_COT, respectively.

**Figure 4:**
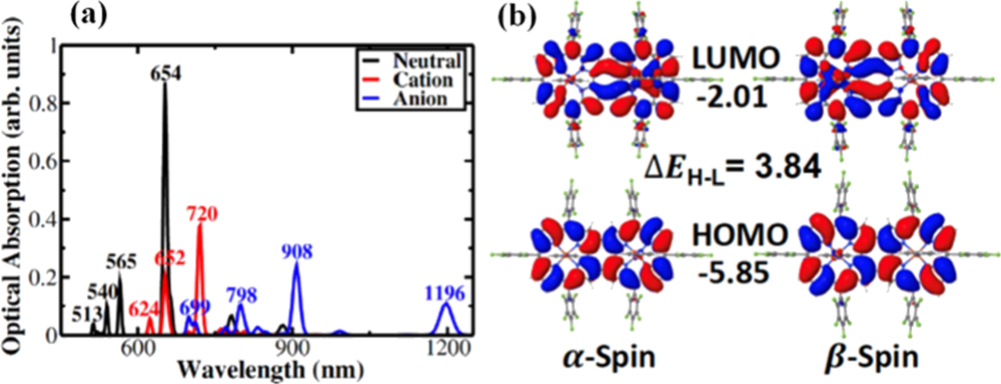
(a) Simulated absorption spectra of neutral (black line), cationic (red line), and anionic (blue line) [Fe^III^tpfc(py)]_2_COT species. (b) HOMO and LUMO iso-surfaces and energies (in eV).

The interaction between the two corrole subunits was further examined and ascertained by UV–Vis spectroscopy and electrochemical techniques. The electronic spectrum of the dimer is very different from that of the monomer (Figure S6); a strongly split Soret band at 385 and 426 nm and three Q-bands at 632, 723, and 798 nm for the former, compared to one Soret band at 406 nm and two Q-bands at 513 and 710 nm for the latter were observed. The calculated low-lying optical absorption peaks (Figure 4a) appear at ∼654, ∼565, and ∼540 nm for the dimer [Fe^III^tpfc(py)]_2_COT, as compared to the corresponding monomer absorption peak at ∼462 nm. This is in reasonable (∼0.2 to 0.3 eV) agreement with the measured peak values (∼723 and ∼632 nm) for the dimer. The quantitative difference is attributed to the presence of the highly polar solvent DMF in the experiment. The calculated data show that the intense visible bands at 600–800 nm, which appear only in the dimer spectrum, are due to a reduced electronic gap owing to an extensive π-electron delocalization through the COT bridge (see highest occupied (HOMO) and least unoccupied molecular orbital (LUMO) distribution in Figure 4b).

**Figure 5:**
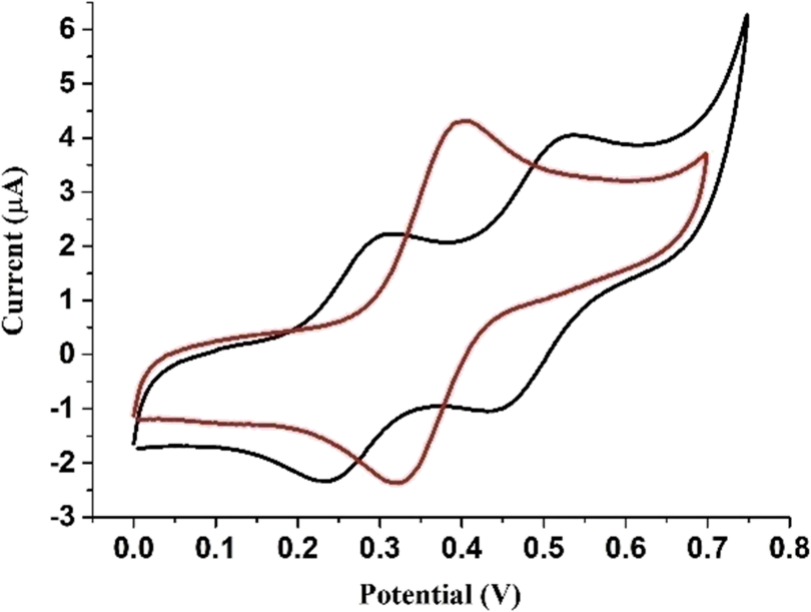
Cyclic voltammograms of Fe^III^tpfc(py) (red) and [Fe^III^tpfc(py)]_2_COT (black), measured in DMF. Conditions: 0.5 mM complex, 0.1 M tetrabutylammonium perchlorate (TBAP), argon saturated, 500 mV/s. Working electrode—glassy carbon, counter electrode—Pt wire, and reference electrode—Ag/AgNO_3_. *E*_1/2_ (ferrocene) = 0.075 V.

Cyclic voltammetry of [Fe^III^tpfc(py)]_2_COT in DMF discloses two reversible oxidation processes with half wave potentials (*E*_1/2_) of 0.27 and 0.50 V, compared to one reversible oxidation process for the monomer, Fe^III^tpfc(py), at *E*_1/2_ = 0.36 V under identical conditions (Figures 5 and S13). The large separation between the two redox processes for the dimer, with the oxidation potential for the monomer being in between them, was previously observed in the cyclic voltammograms of the analogous gallium complexes.^15^ Using a nonredox metal ion such as gallium in the bis-corrole moiety is very convenient, because it serves as a prototype and hence eliminates questions as to the site of the electron transfer. Measuring the redox potential of Ga^III^tpfc(py) (Figure 1) under similar conditions yields a half wave potential of 0.31 V (Figure S14), similar to *E*_1/2_ = 0.36 V of Fe^III^tpfc(py), which suggests that the electron transfer occurs mainly on the corrole macrocycle for the iron(III) corrole dimer as well.

**Figure 6:**
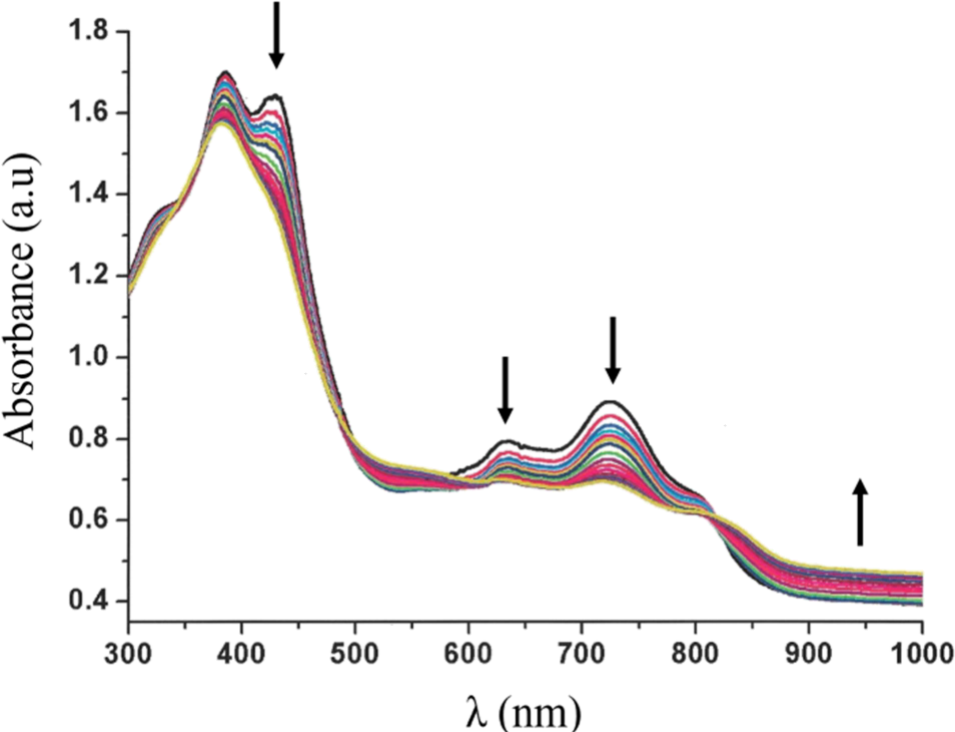
UV–Vis spectral changes during the controlled potential oxidation of 0.25 mM [Fe^III^tpfc(py)]_2_COT in DMF. Conditions: +0.7 V, 0.2 M TBAP, argon saturated. Working electrode—Pt gauze, counter electrode—Pt wire, and reference electrode—Ag/AgNO_3_.

Supporting evidence for this conclusion, as well as additional insights, were obtained by spectroelectrochemistry. Changes in the electronic spectrum of Fe^III^tpfc(py) (Figure S15) after applying a constant potential of +0.6 V are consistent with corrole-centered oxidation, similar to those observed for the redox-inactive analogues in Ga^III^tpfc(py)^32^ and Al^III^tpfc(py)_2_.^33^ The Soret band decreased in intensity and blue-shifted from 406 to 350 nm, the Q band at 513 nm decreased, accompanied by an increase in absorption at the 600–900 nm range. Using a reverse potential of +0.05 V led to complete restoration of the initial spectrum, confirming the reversibility of the redox process seen in cyclic voltammetry. As to the dimer, an anodic potential of +0.7 V after the second redox process led to changes that are similar to those of the monomer: intensity decrease of the Soret and Q-bands and an increase in absorption in the 800–1000 nm region (Figure 6). This suggests that in the case of dimer oxidation, the site of electron transfer is the bis-corrole macrocycle. Optical spectra calculated for the neutral and the oxidized states (Figure 4a) of the dimer [Fe^III^tpfc(py)]_2_COT show that the absorption peak at ∼654 nm in the neutral form is replaced by a less intense peak at ∼652 nm and a red-shifted peak at ∼720 nm for the oxidized state, along with intensified low energy absorption owing to transitions involving the singly occupied molecular orbital. This is consistent with the experimentally observed spectroelectrochemical changes (see Figure 6). To shed further light on the oxidation site, we examined the differences in DFT-computed self-consistent charge densities, between the neutral and the cationic states (Figure S17). We find that the holes are localized indeed on the macrocycle, suggesting a corrole-centered one-electron oxidation process for all studied complexes. This also agrees well with the HOMO distribution shown in Figure 4b.

**Figure 7:**
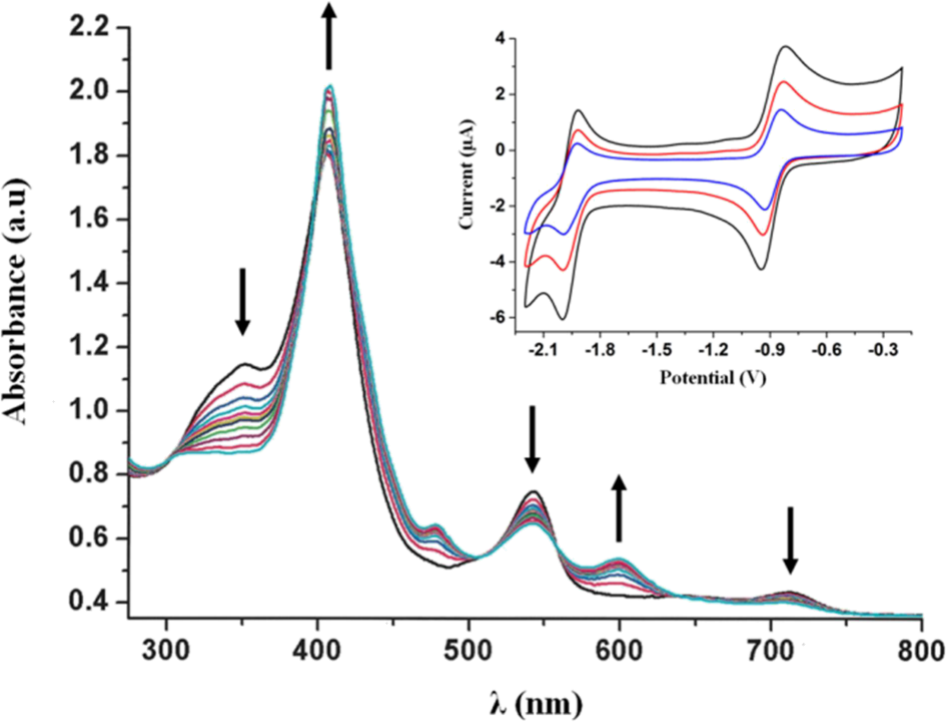
UV–Vis spectral changes during the controlled potential reduction of 0.25 mM Fe^III^tpfc(py) in DMF, 0.2 M TBAP, −1.5 V, argon saturated. Inset: cyclic voltammogram of 0.50 mM Fe^III^tpfc(py) at 100, 250, and 500 mV/s, measured in DMF. 0.1 M TBAP, argon saturated. Conditions: working electrode—glassy carbon/Pt gauze, counter electrode—Pt wire, and reference electrode—Ag/AgNO_3_. *E*_1/2_ (ferrocene) = 0.09 V.

The cyclic voltammetry of Fe^III^tpfc(py) at negative potentials uncovered two reversible redox processes, at *E*_1/2_ = −0.88 V and *E*_1/2_ = −1.95 V (Figure 7, Inset). Under identical conditions, the *E*_1/2_ for Ga^III^tpfc(py) is −1.70 V, which along with *E*_1/2_ = +0.31 V mentioned earlier, corresponds to about a 2 V HOMO–LUMO gap that is typical of nonredox corrole complexes.^34,35^ The smaller difference between the first oxidation and reduction potentials measured for Fe^III^tpfc(py), Δ*E* = 1.24 V, implies the first reduction process to be metal centered, i.e., Fe^III/II^. Applying a constant potential of −1.5 V led to spectral changes which support the formation of [Fe^II^tpfc]^−^: indeed an increase in the intensity of the Soret band at 406 nm accompanied by a clear isosbestic point and formation of a new low intensity Q band at 600 nm (Figure 7) was observed. This is usually assigned to ligand-to-metal charge transfer and not to the formation of the corrole radical anion, where a significant decrease in all the absorption intensities would be expected. Also, the spectrum of electrochemically generated [Fe^II^tpfc(py)]^−^ is very similar to the one chemically generated by reducing Fe^III^tpfc(py) with sodium amalgam in acetonitrile solution.^36^ Using a reverse potential of −0.2 V led to the complete restoration of the initial spectrum, confirming the reversibility of the redox process seen in its CV (Figure S16). Furthermore, the electrochemical reversibility of the first redox process indicates that during the time scale of the cathodic sweep there is no change in the structure of the Fe^III^tpfc(py) complex, i.e., no dissociation of coordinated pyridine.

**Figure 8:**
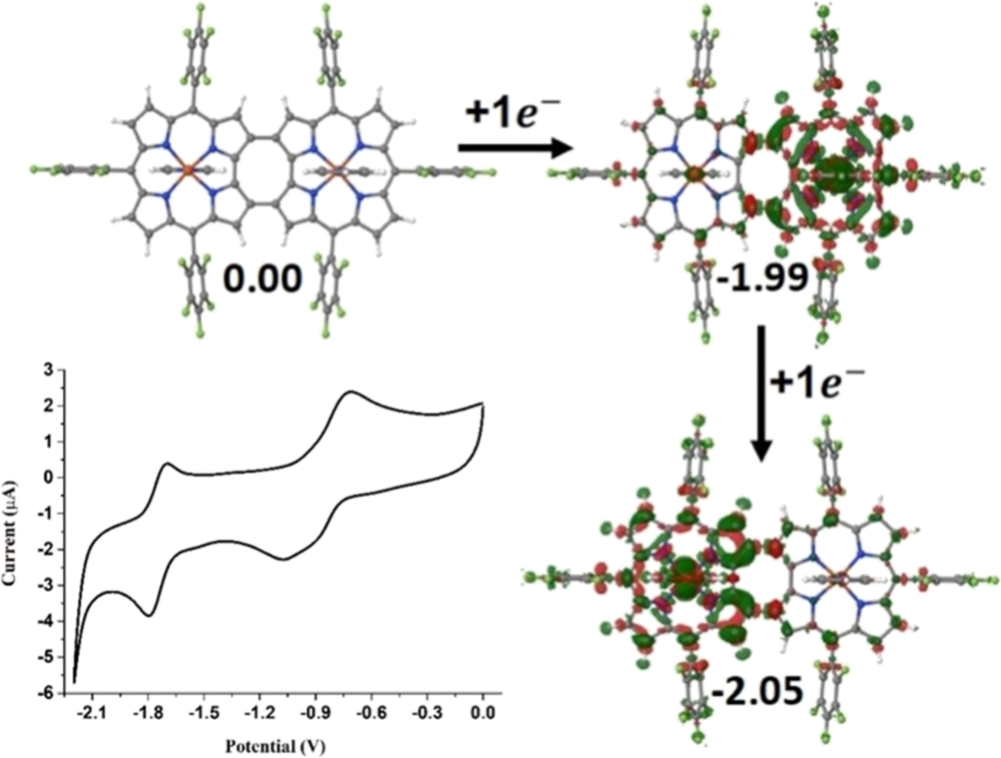
Differences in self-consistent charge density calculated for the COT-fused Fe(III) corrole dimer, relative to the state with one less electron. Iso-surface value of 0.001 electrons/bohr^3^ was used. Electron’s addition and removal energies are in eV. In the lower left side of the figure: Cyclic voltammogram of [Fe^III^tpfc(py)]_2_COT measured in DMF. Conditions: 0.5 mM complex, 0.1 M TBAP, argon saturated, 250 mV/s. Working electrode—glassy carbon, counter electrode—Pt wire, and reference electrode—Ag/AgNO_3_. *E*_1/2_ (ferrocene) = 0.075 V.

A cathodic sweep of the iron corrole dimer yielded a very interesting cyclic voltammogram, exhibiting a reversible reduction process with two poorly separated reduction waves around −1.0 V, followed by an additional reversible redox process at *E*_1/2_ = −1.75 V (Figure 8). Looking at the cyclic voltammogram of the analogous gallium complex at negative potentials revealed a large separation, ≈400 mV, between the ligand centered reductions, which was indicative of efficient conjugation between the two corrole units through the COT moiety. The poorly separated reduction waves, along with the peak area of the first redox process being approximately two times larger than the peak area for the redox process at −1.75 V, suggest two consecutive electron transfer steps to the Fe^III^ centers, indicative of very weak electron communication between the two redox active iron(III) sites. As shown in Figure 8, the calculated charge density differences and electron addition energy for the first and second reduction processes in [Fe^III^tpfc(py)]_2_COT clearly reveal two successive electron transfer steps, occurring mainly at the Fe centers, with similar energetics (≈ −2.0 eV). This supports weak electronic communication between the two redox active Fe^III^ sites in the COT-fused dimer.

Experimental evidence for the above conclusions was looked for by examining the temperature-dependent magnetic susceptibility by the superconducting quantum interference device magnetometry of the 5- and the 6-coordinate complexes. These results provided evidence for two *S* = 3/2 (iron(III), intermediate spin) and two *S* = 1/2 (iron(III), low spin) subunits in [Fe^III^tpfc(py)]_2_COT and [Fe^III^tpfc(py)_2_]_2_COT, respectively (Figure S10). The simulated metal–metal exchange interactions (*J*) for [Fe^III^tpfc(py)]_2_COT and [Fe^III^tpfc(py)_2_]_2_COT at 2 K are −10 and −4 cm^–1^, respectively. These small *J* values reflect weak antiferromagnetic couplings between the two iron metal centers in the corrole dimer. Similar conclusions were derived from the electron paramagnetic resonance (EPR) spectrum of [Fe^III^tpfc(py)_2_]_2_COT at 20 K, recorded in a frozen pyridine/chloroform/toluene solution: characteristic low-spin iron(III) corrole *g* values of *g*_1_ = 1.7, *g*_2_ = 2.0, and *g*_3_ = 2.24 with a line width of 664 G. The EPR spectrum of the Fe^III^tpfc(py)_2_ monomer at the same conditions exhibited a rhombic EPR spectra with *g* values of *g_x_* = 1.800, *g_y_* = 2.208, and *g_z_* = 2.500. This indicates that the spin state of [Fe^III^tpfc(py)_2_]_2_COT can be assigned as two almost isolated low-spin iron(III) corroles at 20 K.

The two Fe^III^ centers were deduced to interact with each other only weakly in the ground electronic state as well, as indicated by the ∼4 to 5 meV energy difference between the ferromagnetic and antiferromagnetic spin configurations. This is also reflected in the well-separated and localized spin density distribution for the complex (Figure S18). A similar symmetrical spin density distribution was found in a neutral homobimetallic porphyrin analogue, bridged by ethylene group.^37^ Computation shows the third and fourth reduction processes to occur at the macrocyclic ring, but with unfavorable electron addition energetics (Figure S17). Spectral changes recorded after applying a constant potential of −1.5 V on [Fe^III^tpfc(py)]_2_COT led to changes in the UV–Vis spectrum that are beyond our ability to analyze at this moment: formation of an absorption band at 750 nm with significant intensity and decrease in intensity of absorption band at 385 nm. An isosbestic point at 545 nm is observed as well (Figure S19).

**Figure 9:**
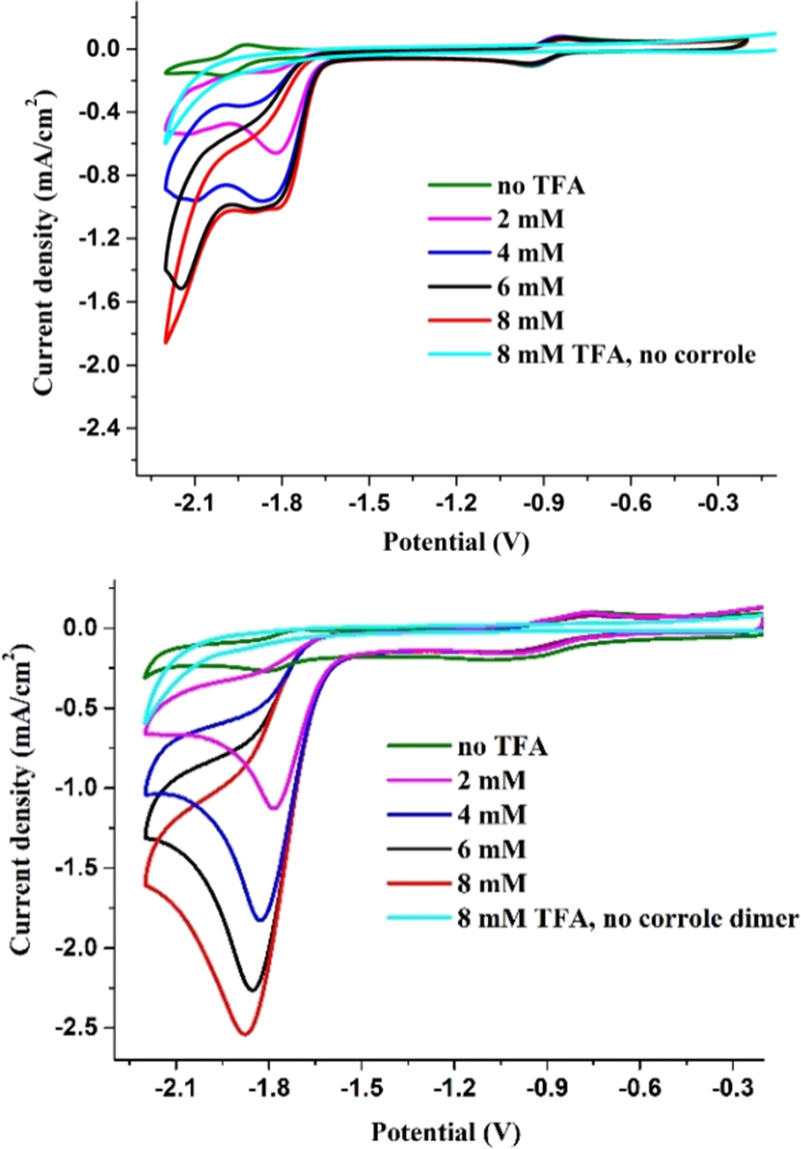
Electrocatalytic proton reduction with Fe^III^tpfc(py) (top) and [Fe^III^tpfc(py)_2_]_2_COT (bottom). 0.5 mM complex, 0.1 M TBAP, argon saturated, 250 mv/s, DMF. [TFA] = 0, 2,4, 6, and 8 mM.

The practical outcome of an additional redox site in the conjugated system was briefly tested by examining the ability of [Fe^III^tpfc(py)]_2_COT to act as an electrocatalyst for proton reduction. One motivation was to test if the catalytic onset potential of the bis-iron(III) complex may occur earlier than for the mononuclear analogue, i.e., after Fe^III^ to Fe^II^ reduction of each metal center in the dimer rather than the two electron reduction of Fe^III^ to Fe^I^ required for the monomer. This question was addressed by measuring the electrochemistry of the Fe^III^ dimer as a function of added acid, compared with results obtained by applying Fe^III^tpfc(py) under the same conditions. The results, shown in Figure 9, indicate that: (a) for both complexes, Fe^III^ monomer and dimer, an increase in trifluoroacetic acid (TFA) concentrations is accompanied by an increase in current and the appearance of an irreversible reduction wave—both indicative of a catalytic proton reduction; (b) catalysis requires reduction beyond the iron(II) oxidation state, in both cases; (c) in the case of the monomer, the increase in current at −1.8 V reaches a plateau after the addition of 4 mM of TFA, while in the case of the dimer the increase in *i*_cat_ values continues beyond that; (d) the catalytic onset potential for the bimetallic complex is about 100 mV earlier than for the monometalic one, similar to the differences in their Fe^I^/Fe^II^ redox potentials in the absence of acid; (e) *i*_cat_ values for the dimer are at least two times higher than *i*_cat_ values for the monomer, for a similar TFA concentration; (f) this also translates to a fourfold difference in reaction rates (110 vs 28 s^–1^), as estimated by using *k*_obs_ = 1.94ν(*i*_cat_/*i*_p_)^2^ for the results with 4 mM TFA. These observations indicate that the additional Fe^III^ corrole unit has a synergistic effect on the catalytic activity, thus justifying additional research dedicated to acquiring an additional insight into this encouraging phenomenon.

## 4 Conclusions

After previously resolving the issue of conjugation between the two corrole subunits via the antiaromatic octatetraene moiety for the nonredox metal centers, in this work, we set out to explore a more challenging question—what is the effect of a redox active metal, in this case Fe(III), on the electronic communication between the two corrole subunits?

Answering this question was definitely more demanding. First, two Fe(III) bis-corrole complexes were isolated and fully characterized: 5-coordinate [Fe^III^tpfc(py)]_2_COT and 6-coordinate [Fe^III^tpfc(py)_2_]_2_COT, with one and two axial pyridine molecules on each metal ion, respectively. Both complexes were then characterized by an array of experimental and theoretical methodologies. X-ray crystallography revealed a dome-shaped structure for [Fe^III^tpfc(py)]_2_COT and a perfectly planar geometry for the analogous bis-pyridine complex, with reduced Fe–N_c_ and Fe–N_py_ distances upon coordination of a second axial pyridine. These results were perfectly aligned with DFT computations, leading to the conclusion that the coordination of a second pyridine molecule leads to the destabilization of the Fe *d*_*x*^2^ – *y*^2^_ orbital and therefore a change in the Fe(III) spin state from intermediate (*S* = 3/2) to low (*S* = 1/2).

Cyclic voltammetry and spectroelectrochemistry, corroborated by DFT computations, shed light on the site of electron transfer. Oxidation creates a vacancy in the π-system that is on the corrole macrocycle, involving conjugation between both corrole subunits; contrarily, reduction is characterized by two consecutive electron transfers to the Fe^III^ centers with similar energetics, suggesting a very weak electron communication between the two redox active iron(III) sites. The effect of an additional redox site has proven to be beneficial for electrocatalytic proton reduction. Ongoing efforts address isolation of reactive intermediates and identification of reaction pathways under both homogeneous and heterogenous reaction conditions.

## Supporting Information Available

^1^H and ^19^F NMR spectra; EPR spectra, mass spectra, magnetic susceptibility, UV/vis, and X-ray analysis; synthetic pathways; optimized geometries; packing diagram; cyclic voltammograms; differences in self-consistent charge density; spectroelectrochemical, electrochemical, and computational data; electronic coupling and electrocatalysis in redox active fused iron corroles (PDF)

## References

[ref1] aOoi S.; Tanaka T.; Osuka A. Metal Complexes of Meso–Meso Linked Corrole Dimers. Inorg. Chem. 2016, 55, 8920–8927. 10.1021/acs.inorgchem.6b01422. 27533780

[ref2] Ooi S.; Tanaka T.; Park K. H.; Lee S.; Kim D.; Osuka A. Fused Corrole Dimers Interconvert between Nonaromatic and Aromatic States through Two-Electron Redox Reactions. Angew. Chem., Int. Ed. 2015, 54, 3107–3111. 10.1002/anie.201411242. 25573778

[ref3] Hazari A. S.; Chandra S.; Kar S.; Sarkar B. Metal Complexes of Singly, Doubly and Triply Linked Porphyrins and Corroles: An Insight into the Physicochemical Properties. Chem. – Eur. J. 2022, 28, e202104550 10.1002/chem.202104550. 35088477 PMC9311859

[ref4] Nagai T.; Takiguchi A.; Ueda M.; Oda K.; Hiroto S.; Shinokubo H. X-Shaped Cyclobutane-Linked Tetraporphyrins through a Thermal [2+2] Cycloaddition of Etheno-Fused Diporphyrins. J. Am. Chem. Soc. 2018, 140, 8392–8395. 10.1021/jacs.8b04673. 29925230

[ref5] Shimizu D.; Osuka A. Porphyrinoids as a Platform of Stable Radicals. Chem. Sci. 2018, 9, 1408–1423. 10.1039/C7SC05210C. 29675188 PMC5892410

[ref6] Tsuda A.; Osuka A. Fully Conjugated Porphyrin Tapes with Electronic Absorption Bands That Reach into Infrared. Science 2001, 293, 79–82. 10.1126/science.1059552. 11441176

[ref7] Tsuda A.; Furuta H.; Osuka A. Syntheses, Structural Characterizations, and Optical and Electrochemical Properties of Directly Fused Diporphyrins. J. Am. Chem. Soc. 2001, 123, 10304–10321. 10.1021/ja0110933. 11603981

[ref8] Ooi S.; Shimizu D.; Furukawa K.; Tanaka T.; Osuka A. Stable Face-to-Face Singlet Diradicaloids: Triply Linked Corrole Dimer Gallium(III) Complexes with Two μ-Hydroxo-Bridges. Angew. Chem., Int. Ed. 2018, 57, 14916–14920. 10.1002/anie.201810200. 30239078

[ref9] Sil D.; Dey S.; Kumar A.; Bhowmik S.; Rath S. P. Oxidation Triggers Extensive Conjugation and Unusual Stabilization of Two Di-Heme Dication Diradical Intermediates: Role of Bridging Group for Electronic Communication. Chem. Sci. 2016, 7, 1212–1223. 10.1039/C5SC03120F. 29910877 PMC5975787

[ref10] Tait C. E.; Neuhaus P.; Anderson H. L.; Timmel C. R. Triplet State Delocalization in a Conjugated Porphyrin Dimer Probed by Transient Electron Paramagnetic Resonance Techniques. J. Am. Chem. Soc. 2015, 137, 6670–6679. 10.1021/jacs.5b03249. 25914154 PMC4569061

[ref11] Ooi S.; Ueta K.; Tanaka T.; Osuka A. Singly, Doubly, and Triply Linked Corrole Oligomers: Synthesis, Structures, and Linking Position Dependent Properties. ChemPlusChem 2019, 84, 578–588. 10.1002/cplu.201800570. 31944025

[ref12] Cho S.; Lim J. M.; Hiroto S.; Kim P.; Shinokubo H.; Osuka A.; Kim D. Unusual Interchromophoric Interactions in β,Β′ Directly and Doubly Linked Corrole Dimers: Prohibited Electronic Communication and Abnormal Singlet Ground States. J. Am. Chem. Soc. 2009, 131, 6412–6420. 10.1021/ja900220y. 19378950

[ref13] Hiroto S.; Furukawa K.; Shinokubo H.; Osuka A. Synthesis and Biradicaloid Character of Doubly Linked Corrole Dimers. J. Am. Chem. Soc. 2006, 128, 12380–12381. 10.1021/ja062654z. 16984164

[ref14] Tanaka T.; Osuka A. Triply Linked Porphyrinoids. Chem. – Eur. J. 2018, 24, 17188–17200. 10.1002/chem.201802810. 29943429

[ref15] Bhowmik S.; Kosa M.; Mizrahi A.; Fridman N.; Saphier M.; Stanger A.; Gross Z. The Planar Cyclooctatetraene Bridge in Bis-Metallic Macrocycles: Isolating or Conjugating?. Inorg. Chem. 2017, 56, 2287–2296. 10.1021/acs.inorgchem.6b02944. 28182414

[ref16] Simkhovich L.; Mahammed A.; Goldberg I.; Gross Z. Synthesis and Characterization of Germanium, Tin, Phosphorus, Iron, and Rhodium Complexes of Tris(Pentafluorophenyl)Corrole, and the Utilization of the Iron and Rhodium Corroles as Cyclopropanation Catalysts. Chem. – Eur. J. 2001, 7, 1041–1055. 10.1002/1521-3765(20010302)7:5<1041::AID-CHEM1041>3.0.CO;2-8. 11303864

[ref17] Van Caemelbecke E.; Will S.; Autret M.; Adamian V. A.; Lex J.; Gisselbrecht J.-P.; Gross M.; Vogel E.; Kadish K. M. Electrochemical and Spectral Characterization of Iron Corroles in High and Low Oxidation States: First Structural Characterization of an Iron(IV) Tetrapyrrole π Cation Radical. Inorg. Chem. 1996, 35, 184–192. 10.1021/ic9509037. 11666183

[ref18] Vogel E.; Will S.; Tilling A. S.; Neumann L.; Lex J.; Bill E.; Trautwein A. X.; Wieghardt K. Metallocorroles with Formally Tetravalent Iron. Angew. Chem., Int. Ed. Engl. 1994, 33, 731–735. 10.1002/anie.199407311.

[ref19] Barata J. F. B.; Silva A. M. G.; Neves M. G. P. M. S.; Tomé A. C.; Silva A. M. S.; Cavaleiro J. A. S. β,Β′–Corrole Dimers. Tetrahedron Lett. 2006, 47, 8171–8174. 10.1016/j.tetlet.2006.09.026.

[ref20] Shao Y.; Gan Z.; Epifanovsky E.; Gilbert A. T. B.; Wormit M.; Kussmann J.; Lange A. W.; Behn A.; Deng J.; Feng X.; Ghosh D.; Goldey M.; Horn P. R.; Jacobson L. D.; Kaliman I.; Khaliullin R. Z.; Kuś T.; Landau A.; Liu J.; Proynov E. I.; Rhee Y. M.; Richard R. M.; Rohrdanz M. A.; Steele R. P.; Sundstrom E. J.; Woodcock H. L.; Zimmerman P. M.; Zuev D.; Albrecht B.; Alguire E.; Austin B.; Beran G. J. O.; Bernard Y. A.; Berquist E.; Brandhorst K.; Bravaya K. B.; Brown S. T.; Casanova D.; Chang C.-M.; Chen Y.; Chien S. H.; Closser K. D.; Crittenden D. L.; Diedenhofen M.; DiStasio R. A.; Do H.; Dutoi A. D.; Edgar R. G.; Fatehi S.; Fusti-Molnar L.; Ghysels A.; Golubeva-Zadorozhnaya A.; Gomes J.; Hanson-Heine M. W. D.; Harbach P. H. P.; Hauser A. W.; Hohenstein E. G.; Holden Z. C.; Jagau T.-C.; Ji H.; Kaduk B.; Khistyaev K.; Kim J.; Kim J.; King R. A.; Klunzinger P.; Kosenkov D.; Kowalczyk T.; Krauter C. M.; Lao K. U.; Laurent A. D.; Lawler K. V.; Levchenko S. V.; Lin C. Y.; Liu F.; Livshits E.; Lochan R. C.; Luenser A.; Manohar P.; Manzer S. F.; Mao S.-P.; Mardirossian N.; Marenich A. V.; Maurer S. A.; Mayhall N. J.; Neuscamman E.; Oana C. M.; Olivares-Amaya R.; O’Neill D. P.; Parkhill J. A.; Perrine T. M.; Peverati R.; Prociuk A.; Rehn D. R.; Rosta E.; Russ N. J.; Sharada S. M.; Sharma S.; Small D. W.; Sodt A.; Stein T.; Stück D.; Su Y.-C.; Thom A. J. W.; Tsuchimochi T.; Vanovschi V.; Vogt L.; Vydrov O.; Wang T.; Watson M. A.; Wenzel J.; White A.; Williams C. F.; Yang J.; Yeganeh S.; Yost S. R.; You Z.-Q.; Zhang I. Y.; Zhang X.; Zhao Y.; Brooks B. R.; Chan G. K. L.; Chipman D. M.; Cramer C. J.; Goddard W. A.; Gordon M. S.; Hehre W. J.; Klamt A.; Schaefer H. F.; Schmidt M. W.; Sherrill C. D.; Truhlar D. G.; Warshel A.; Xu X.; Aspuru-Guzik A.; Baer R.; Bell A. T.; Besley N. A.; Chai J.-D.; Dreuw A.; Dunietz B. D.; Furlani T. R.; Gwaltney S. R.; Hsu C.-P.; Jung Y.; Kong J.; Lambrecht D. S.; Liang W.; Ochsenfeld C.; Rassolov V. A.; Slipchenko L. V.; Subotnik J. E.; Van Voorhis T.; Herbert J. M.; Krylov A. I.; Gill P. M. W.; Head-Gordon M. Advances in Molecular Quantum Chemistry Contained in the Q-Chem 4 Program Package. Mol. Phys. 2015, 113, 184–215. 10.1080/00268976.2014.952696.

[ref21] Chai J.-D.; Head-Gordon M. Long-Range Corrected Hybrid Density Functionals with Damped Atom–Atom Dispersion Corrections. Phys. Chem. Chem. Phys. 2008, 10, 6615–6620. 10.1039/B810189B. 18989472

[ref22] Berryman V. E. J.; Boyd R. J.; Johnson E. R. Balancing Exchange Mixing in Density-Functional Approximations for Iron Porphyrin. J. Chem. Theory Comput. 2015, 11, 3022–3028. 10.1021/acs.jctc.5b00203. 26575739

[ref23] Kronik L.; Stein T.; Refaely-Abramson S.; Baer R. Excitation Gaps of Finite-Sized Systems from Optimally Tuned Range-Separated Hybrid Functionals. J. Chem. Theory Comput. 2012, 8, 1515–1531. 10.1021/ct2009363. 26593646

[ref24] Rohrdanz M. A.; Martins K. M.; Herbert J. M. A Long-Range-Corrected Density Functional That Performs Well for Both Ground-State Properties and Time-Dependent Density Functional Theory Excitation Energies, Including Charge-Transfer Excited States. J. Chem. Phys. 2009, 130, 054112 10.1063/1.3073302. 19206963

[ref25] Egger D. A.; Weissman S.; Refaely-Abramson S.; Sharifzadeh S.; Dauth M.; Baer R.; Kümmel S.; Neaton J. B.; Zojer E.; Kronik L. Outer-Valence Electron Spectra of Prototypical Aromatic Heterocycles from an Optimally Tuned Range-Separated Hybrid Functional. J. Chem. Theory Comput. 2014, 10, 1934–1952. 10.1021/ct400956h. 24839410 PMC4020925

[ref26] Brumboiu I. E.; Prokopiou G.; Kronik L.; Brena B. Valence Electronic Structure of Cobalt Phthalocyanine from an Optimally Tuned Range-Separated Hybrid Functional. J. Chem. Phys. 2017, 147, 044301 10.1063/1.4993623. 28764387

[ref27] Prokopiou G.; Kronik L. Spin-State Energetics of Fe Complexes from an Optimally Tuned Range-Separated Hybrid Functional. *Chemistry – A*. Eur. J. 2018, 24, 5173–5182. 10.1002/chem.201704014. 28984392

[ref28] Simkhovich L.; Goldberg I.; Gross Z. Iron(III) and Iron(IV) Corroles: Synthesis, Spectroscopy, Structures, and No Indications for Corrole Radicals. Inorg. Chem. 2002, 41, 5433–5439. 10.1021/ic020118b. 12377038

[ref29] Munro O. Q.; Serth-Guzzo J. A.; Turowska-Tyrk I.; Mohanrao K.; Shokhireva T. K.; Walker F. A.; Debrunner P. G.; Scheidt W. R. Two Crystalline Forms of Low-Spin [Fe(TMP)(5-MeHIm) _2_ ]ClO _4_ . Relative Parallel and Perpendicular Axial Ligand Orientations. J. Am. Chem. Soc. 1999, 121, 11144–11155. 10.1021/ja991551w.

[ref30] Kadish K. M.; Bottomley L. A.; Beroiz D. Reactions of Pyridine with a Series of Para-Substituted Tetraphenylporphyrincobalt and -Iron Complexes. Inorg. Chem. 1978, 17, 1124–1129. 10.1021/ic50183a006.

[ref31] Nesset M. J. M.; Shokhirev N. V.; Enemark P. D.; Jacobson S. E.; Walker F. A. Models of the Cytochromes. Redox Properties and Thermodynamic Stabilities of Complexes of “Hindered” Iron(III) and Iron(II) Tetraphenylporphyrinates with Substituted Pyridines and Imidazoles. Inorg. Chem. 1996, 35, 5188–5200. 10.1021/ic960491h.

[ref32] Bendix J.; Dmochowski I. J.; Gray H. B.; Mahammed A.; Simkhovich L.; Gross Z. Structural, Electrochemical, and Photophysical Properties of Gallium(Iii) 5,10,15- Tris(Pentafluorophenyl)Corrole. Angew. Chem., Int. Ed. 2000, 39, 4048–4051. 10.1002/1521-3773(20001117)39:22<4048::AID-ANIE4048>3.0.CO;2-7. 11093200

[ref33] Sinha W.; Mizrahi A.; Mahammed A.; Tumanskii B.; Gross Z. Reactive Intermediates Involved in Cobalt Corrole Catalyzed Water Oxidation (and Oxygen Reduction). Inorg. Chem. 2018, 57, 478–485. 10.1021/acs.inorgchem.7b02696. 29256608

[ref34] Mahammed A.; Tumanskii B.; Gross Z. Effect of Bromination on the Electrochemistry, Frontier Orbitals, and Spectroscopy of Metallocorroles. J. Porphyrins Phthalocyanines 2011, 15, 1275–1286. 10.1142/S1088424611004191.

[ref35] Fang Y.; Ou Z.; Kadish K. M. Electrochemistry of Corroles in Nonaqueous Media. Chem. Rev. 2017, 117, 3377–3419. 10.1021/acs.chemrev.6b00546. 28009499

[ref36] Grodkowski J.; Neta P.; Fujita E.; Mahammed A.; Simkhovich L.; Gross Z. Reduction of Cobalt and Iron Corroles and Catalyzed Reduction of CO_2_. J. Phys. Chem. A 2002, 106, 4772–4778. 10.1021/jp013668o.

[ref37] Kumar A.; Usman M.; Samanta D.; Rath S. P. Through Bridge Spin Coupling in Homo- and Hetero-bimetallic Porphyrin Dimers upon Stepwise Oxidations: A Spectroscopic and Theoretical Investigation. Chem. – Eur. J. 2021, 27, 11428–11441. 10.1002/chem.202101384. 34061401

