# Supplementary material for: Electronic Coupling and Electrocatalysis in Redox Active Fused Iron Corroles
Source: Inorg Chem. 2022 Dec 13;61(51):20725–33. doi: 10.1021/acs.inorgchem.2c01389 (PMC9799712; doi:10.1021/acs.inorgchem.2c01389)
Supplement: Supplementary file 1 — ic2c01389_si_001.pdf [file ic2c01389_si_001.pdf]

Supporting Information

Electronic Coupling and Electrocatalysis in Redox active Fused Iron Corroles

Amir Mizrahi,[a,b] Susovan Bhowmik,[b,c] Arun K. Manna,[d] Woormileela Sinha,[b,e] Amit Kumar,[b] Magal Saphier,[a] Atif Mahammed,[b] Moumita Patra,[f] Natalia Fridman,[b] Israel Zilbermann,*[a,g] Leeor Kronik,*[d] and Zeev Gross*[b]

[a] Chemistry Department, Nuclear Research Centre Negev, Beer-Sheva, Israel

[b] Schulich Faculty of Chemistry, Technion-Israel Institute of Technology, Haifa, Israel

[c] Bankura Sammilani College (W.B), India

[d] Department of Molecular Chemistry and Materials Science, Weizmann Institute of Science, Rehovot 76100, Israel

[e] Department of Chemistry, BITS Pilani K K Birla Goa Campus, NH17B, Zuarinagar, Goa-403726, India

[f] Kazi Nazrul University (W.B), India

[g] Chemistry Department, Ben-Gurion University of the Negev, Beer-Sheva, Israel

Corresponding Authors:

*Zeev Gross, E-mail: chr10zg@technion.ac.il, ORCID: 0000-0003-1170-2115

*Israel Zilbermann, E-mail: israelz2003@gmail.com

*Leeor Kronik, E-mail: leeor.kronik@weizmann.ac.il

Table of Contents	Page
Methods and materials	S3
**Figure S1**: NMR spectra of [Fe^{III}tpfc(py)]$_2$COT in $CDCl_3$	S4
**Figure S2**: NMR spectra of [Fe^{III}tpfc(py)$_2$]$_2$COT in pyridine-d_5.	S5
**Figure S3**: NMR spectra of [Fe^{III}tpfc(py)]$_2$COT in toluene-d_8.	S6
**Figure S4**: Curie-plots of ^{1}H NMR signals of the β-pyrrole protons of [Fe^{III}tpfc(py)]$_2$COT in toluene-d_8.	S7
**Figure S5**: Curie-plots of ^{1}H NMR signals of the β-pyrrole protons of [Fe^{III}tpfc(py)$_2$]$_2$COT in pyridine-d_5.	S7
**Figure S6**: UV-Vis spectra of Fe^{III}tpfc(py) and [Fe^{III}tpfc(py)]$_2$COT in DMF.	S8
**Figure S7**: UV-Vis spectra of [Fe^{III}tpfc(py)]$_2$COT in CH_2Cl_2 (blue) and in pyridine (red) at 295K.	S8
**Figure S8**: MS^+ (TOF) of [Fe^{III}tpfc(py)]$_2$COT.	S9
**Figure S9**: The EPR of (a) Fe^{III}tpfc(py)$_2$ and (b) [Fe^{III}tpfc(py)$_2$]$_2$COT (1:1:1 benzene:$CHCl_3$:pyridine; 20K).	S10

**Figure S10**: Variable temperature magnetic susceptibility measurements in the range of 2-300K of $[Fe^{III}tpfc(py)]_2COT$ and $[Fe^{III}tpfc(py)_2]_2COT$.	S11
**Scheme S1**: Synthetic pathways for $[Fe^{III}tpfc(Py)]_2COT$ and $[Fe^{III}tpfc(Py)_2]_2COT$.	S12
**Figure S11:** Optimized geometries of (a) the $[Fe^{III}tpfc(py)]_2COT$ complex, with the Fe(III) being 0.27 Å above the macrocyclic ring, forming a domed-shaped structure, and (b) the planar $[Fe^{III}tpfc(py)_2]_2COT$ complex.	S12
**Figure S12**: Packing diagram of $[Fe^{III}tpfc(py)]_2COT$	S13
**Figure S13**: Cyclic voltammograms of $Fe^{III}tpfc(py)$ and $[Fe^{III}tpfc(py)]_2COT$	S14
**Figure S14**: Cyclic voltammogram of $Ga^{III}tpfc(py)$	S15
**Figure S15**: UV-Vis spectral changes of $Fe^{III}tpfc(py)$ during controlled potential oxidation at +0.6 V	S15
**Figure S16**: Re-oxidation of $[Fe^{II}tpfc(py)]^-$ during controlled potential oxidation at -0.2 V	S16
**Figure S17**: Differences in self-consistent charge-density, calculated for the COT-fused Fe (III) corrole dimer	S16
**Figure S18**: Spin-density distribution of the antiferromagnetic configuration.	S17
**Figure S19**: Spectroelectrochemistry of $[Fe^{III}tpfc(py)]_2COT$.	S17
**Table S1**: Structural parameters for the bis-corrole dimers and monomeric iron corroles analogs.	S13
**Table S2**: Comparison of the C-C bond lengths within the COT moiety as determined by X-ray crystallography.	S14

Methods and materials:

^{1}H and ^{19}F NMR spectra at room temperature were measured using a Bruker Avance III 400 spectrometer equipped with a 5 mm automated tuning and matching broad band probe (BBFO) with z-gradients, operating at 400.4 MHz for ^{1}H and 376.7 for ^{19}F, respectively. Chemical shifts are reported in ppm relative to residual hydrogen atoms in the deuterated solvents $CDCl_3$, toluene-d_8, pyridine-d_5, and DMF-d_5.

Absorption spectra of the samples were measured using an HP 8453 diode array spectrometer. Single crystals immersed in Paratone-N oil were quickly fished with a glass rod and mounted on a Kappa CCD diffractometer under a cold stream of nitrogen at 200 K. Data collection was carried out with mono chromated Mo Kα radiation using φ and ω scans to cover the Ewald sphere. Accurate cell parameters were obtained with complete collections of intensities, and these were corrected in the usual way. The structure was solved by SHELXS-97 direct methods3 and refined by the SHELXL-97 program package. The atoms were refined anisotropically. Hydrogen atoms were calculated using the riding model. The software used for molecular graphics was Mercury 3.1.4.

Cyclic voltammetry measurements were carried out in DMF containing 0.5 mM of the complex and 0.1 M TBAP (Fluka, for electrochemical analysis) as the electrolyte at several scan rates under an argon atmosphere. A conventional three electrode system consisting of a glassy carbon as working electrode, a Pt wire as counter electrode and Ag wire separated from the bulk solution by a sample holder with a porous glass frit in 0.1 M TBAP / 0.01 M $AgNO_3$ as reference electrode. Electrochemical measurements were recorded with an EmStat3+ electrochemical system. All potentials are referenced vs. Ag/$AgNO_3$ and Ferrocene was added as an internal standard, $E_{1/2}$ ($Fc^{+/0}$) = 0.09 or 0.075 V vs. Ag/Ag^{+} in 0.1 M TBAP / 0.01 M $AgNO_3$ solution. For spectroelectrochemistry, the same setup was used, but with platinum gauze as the working electrode, usually at 0.2 MTBAP and 0.25 mM complex concentration. The potential was applied by EmStat3+ electrochemical system and the spectral changes were recorded by an Agilent HP 8453 UV-Visible Spectrophotometer.

Magnetic susceptibility data were collected using a Quantum Design MPMS3 SQUID magnetometer over the temperature range 2 to 300 K. Data were collected over the temperature range of 2 to 300 K, by using applied magnetic fields of 1 T and corrected for diamagnetism using Pascal's constant.[1]

The X-band CW-EPR spectra at low temperature were recorded on a Bruker Elexsys-500 X-band spectrometer using a standard rectangular Bruker EPR cavity (ER 4119 HS) equipped with an Oxford helium cryostat (ESR900) having a continuous flow of liquid helium. The samples were input into a capillary micropipette (BRAND GMBH, Germany) sealed at one end and inserted into an outer quartz tube of 3mm (ID) .Then the tubes were put into the cryostat and measured.

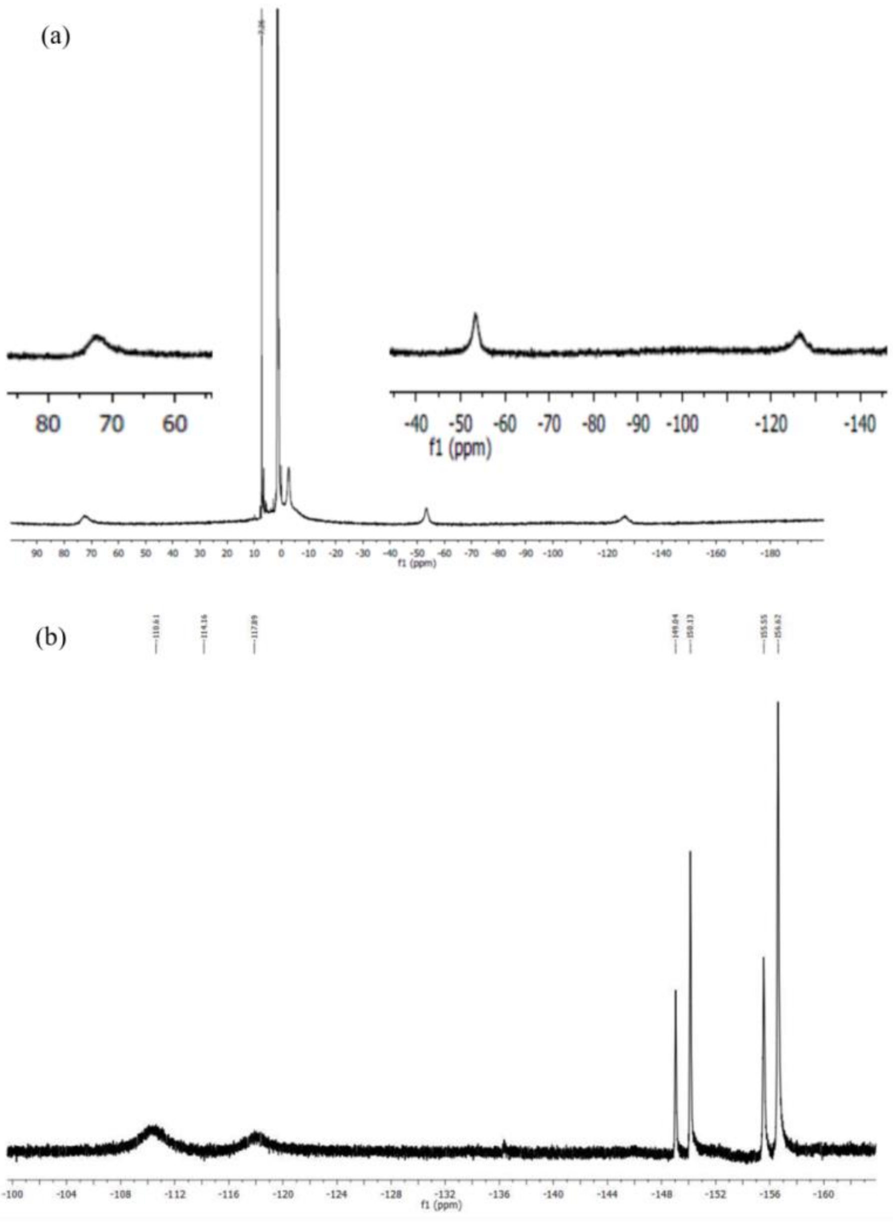

Figure S1: NMR spectra of [Fe^{III}tpfc(py)]$_2$COT in $CDCl_3$, 400 MHz. (a) ^{1}H-NMR. (b) ^{19}F-NMR.

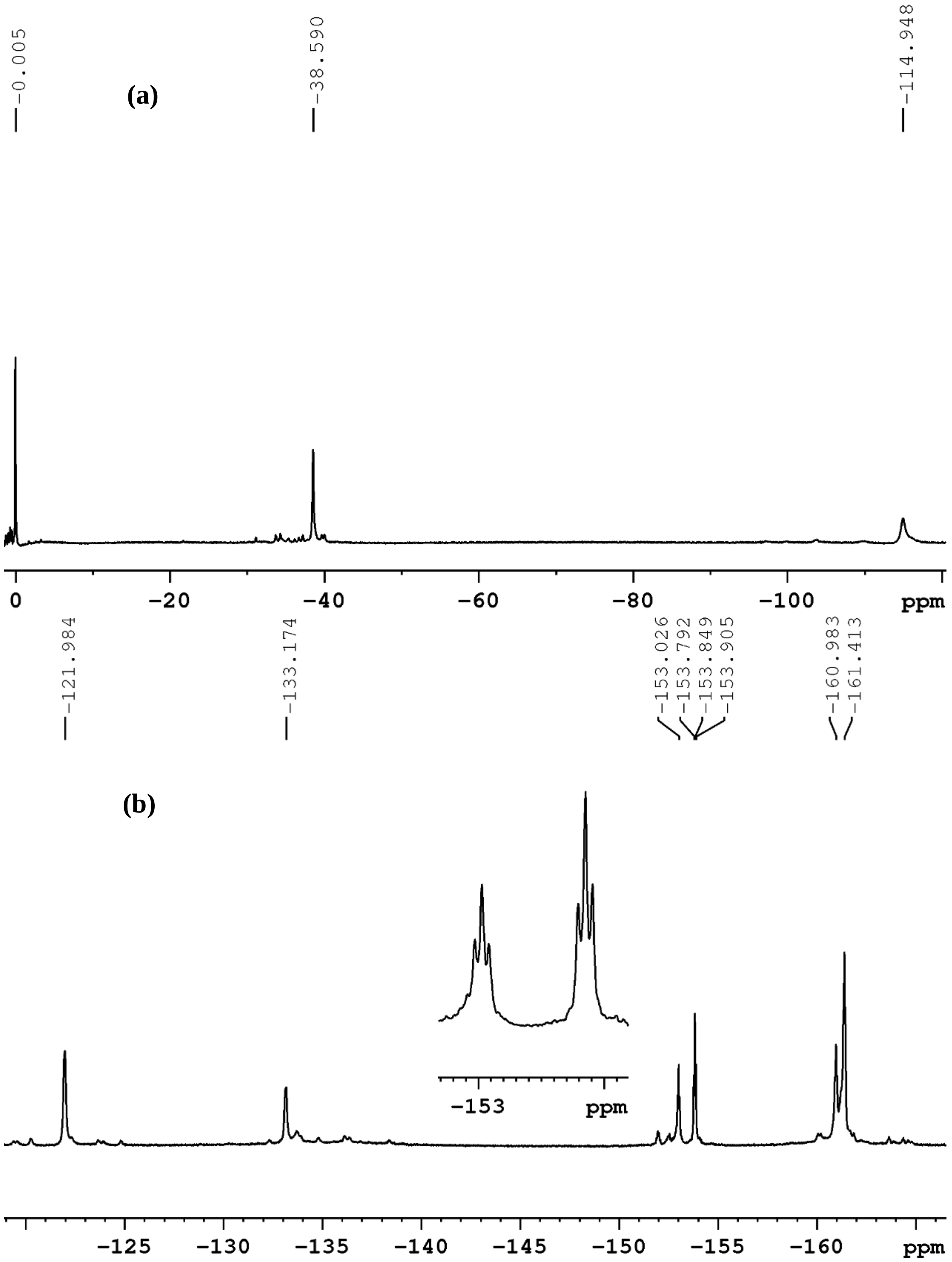

Figure S2: NMR spectra of $[Fe^{III}tpfc(py)_2]_2COT$ in pyridine-d_5, 400 MHz. (a) ^1H-NMR. (b) ^{19}F-NMR.

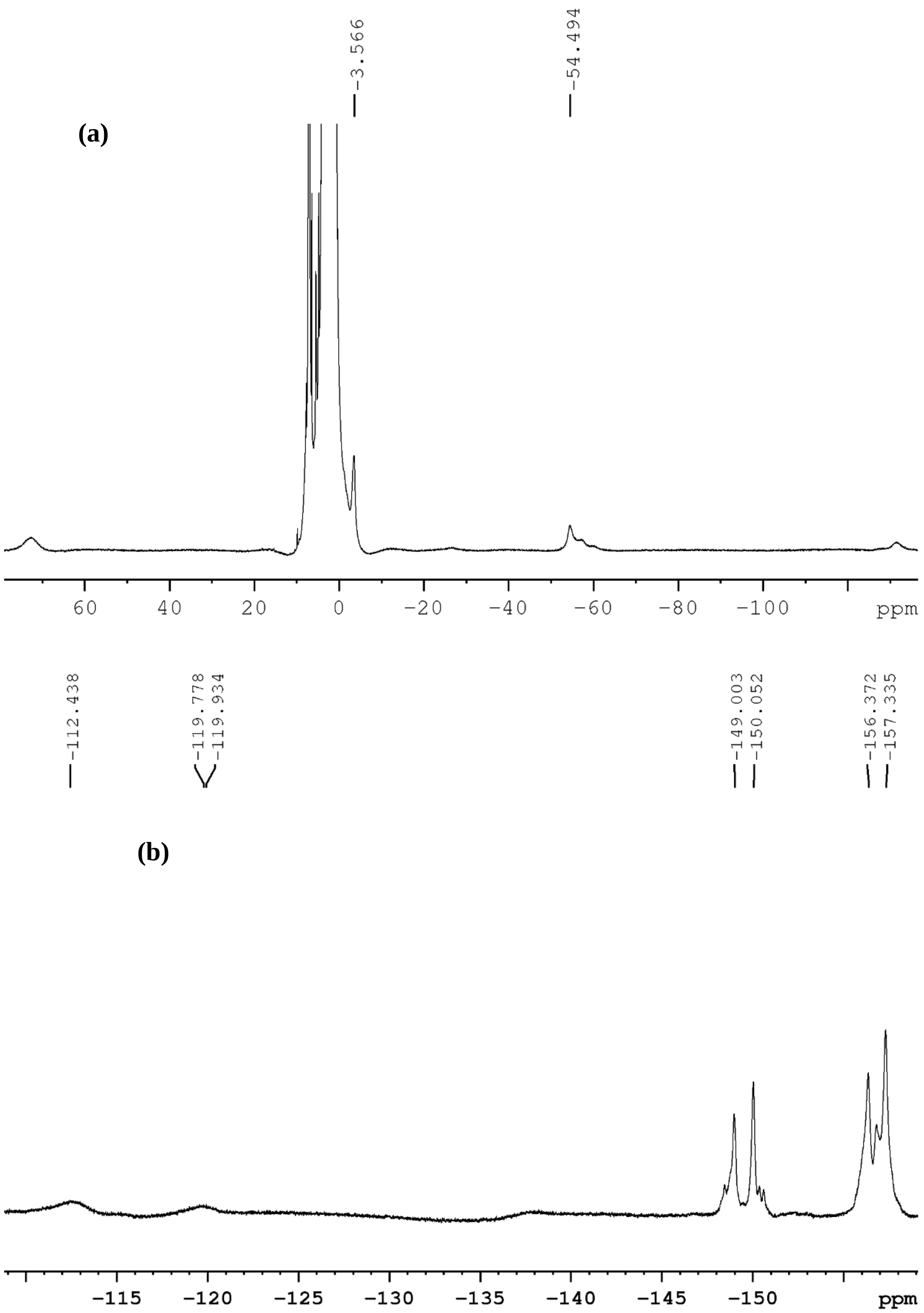

Figure S3: NMR spectra of $[Fe^{III}tpfc(py)]_2COT$ in toluene-d_8, 400 MHz. (a) ^{1}H-NMR. (b) ^{19}F-NMR.

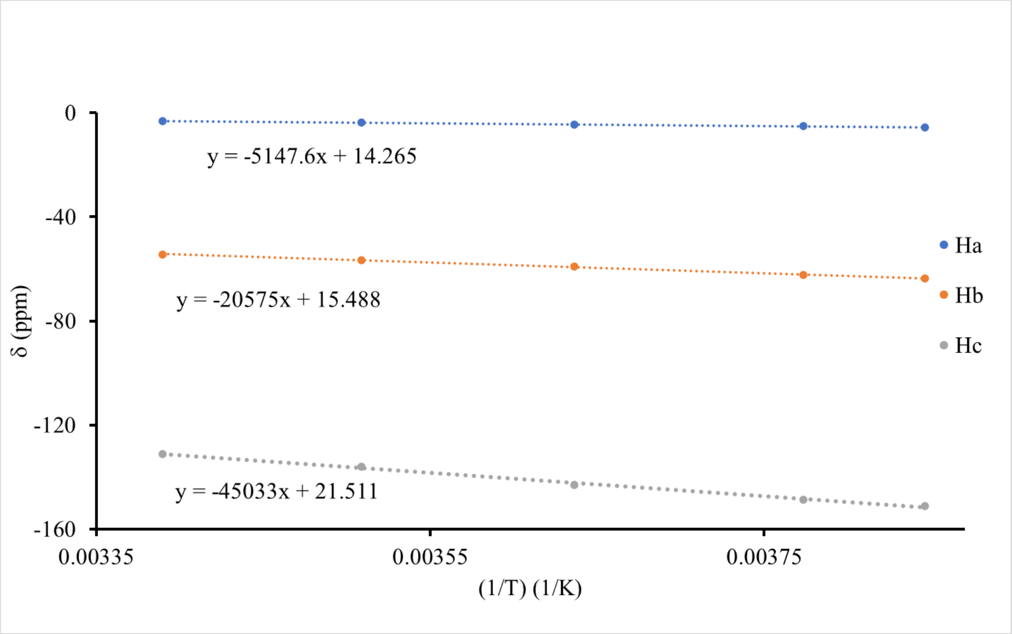

Figure S4: Curie-plots of ^{1}H NMR signals of the β-pyrrole protons of $[Fe^{III}tpfc(py)]_2COT$ in toluene-d_8.

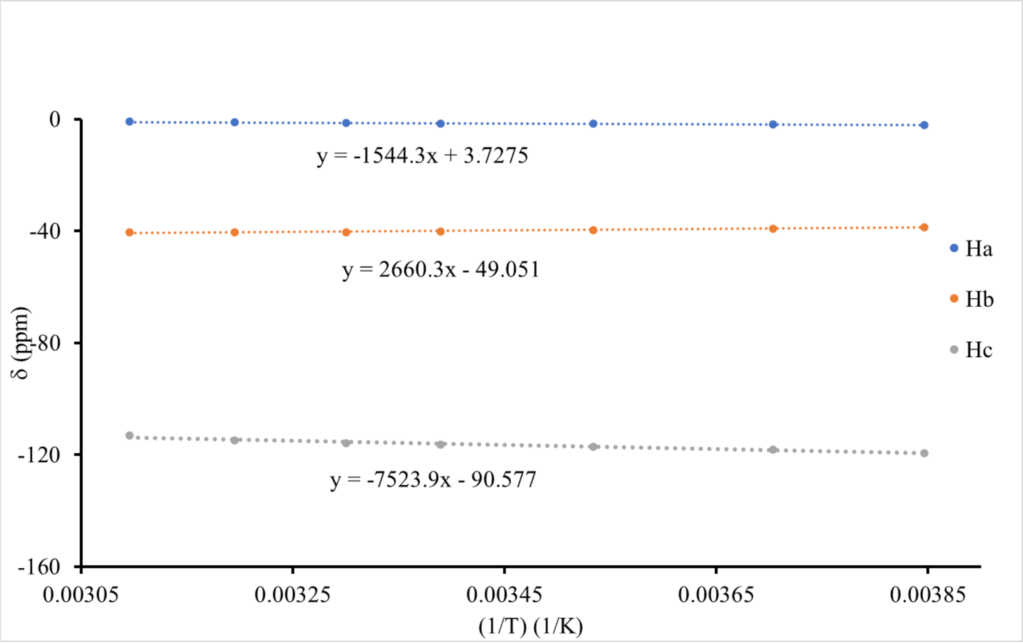

Figure S5: Curie-plots of ^{1}H NMR signals of the β-pyrrole protons of $[Fe^{III}tpfc(py)_2]_2COT$ in pyridine-d_5.

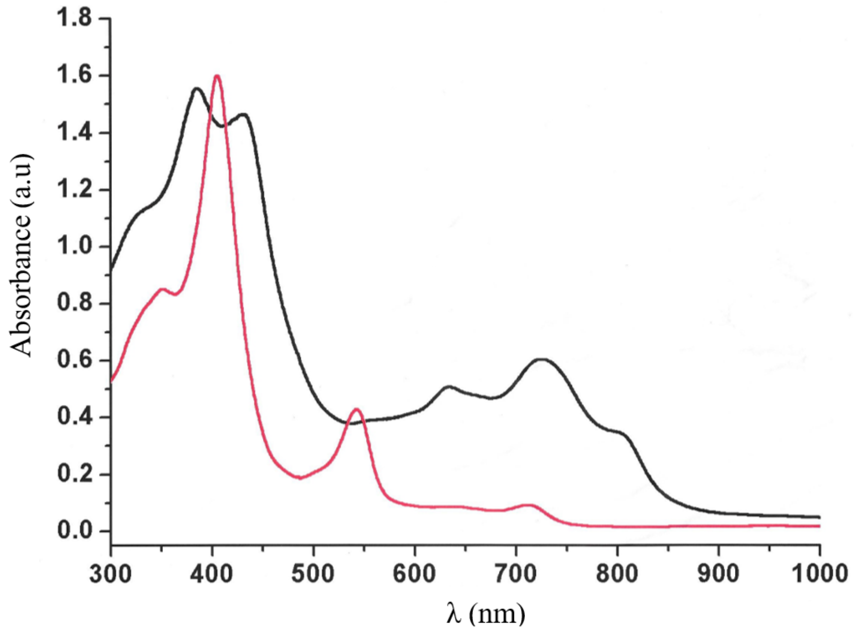

Figure S6: UV-Vis spectra of 26 µM Fe^{III}tpfc(py) (red) and 100 µM [Fe^{III}tpfc(py)]$_2$COT (black) in DMF.

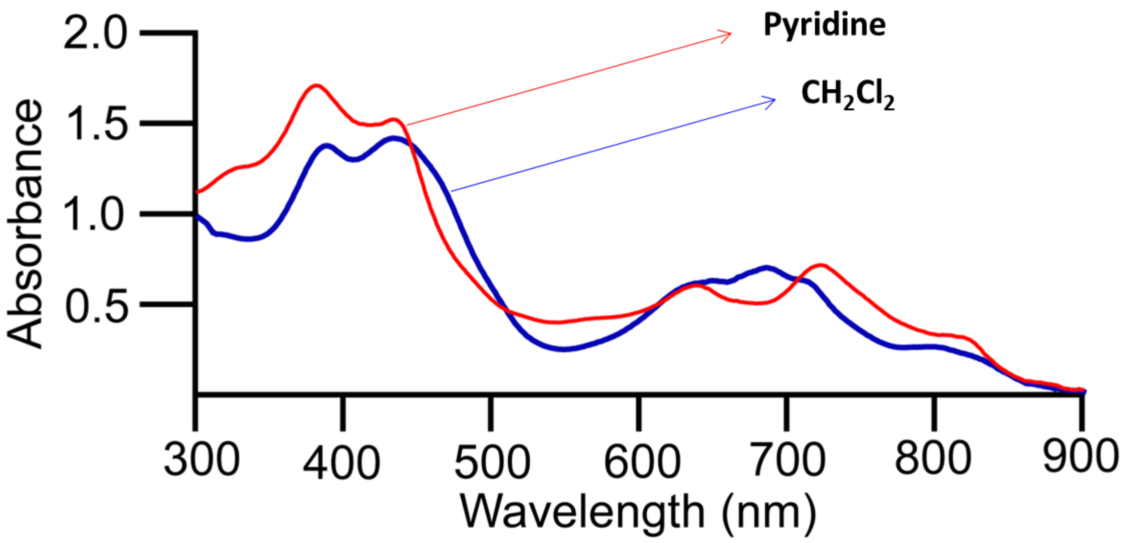

Figure S7: UV-Vis spectra of [Fe^{III}tpfc(py)]$_2$COT in CH_2Cl_2 (blue) and in pyridine (red) at 295K.

[Fe^{III}tpfc(py)]$_2$COT-2py

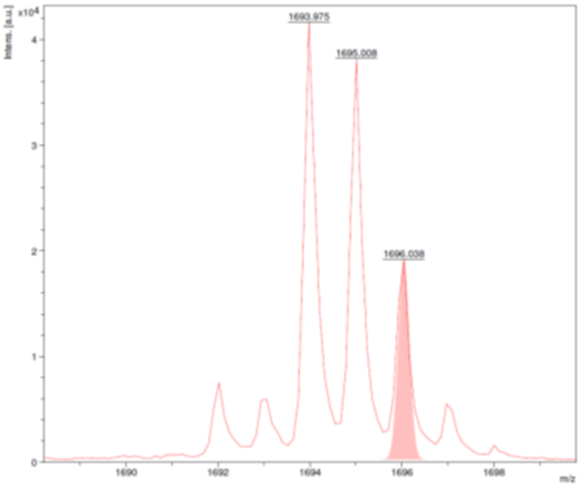

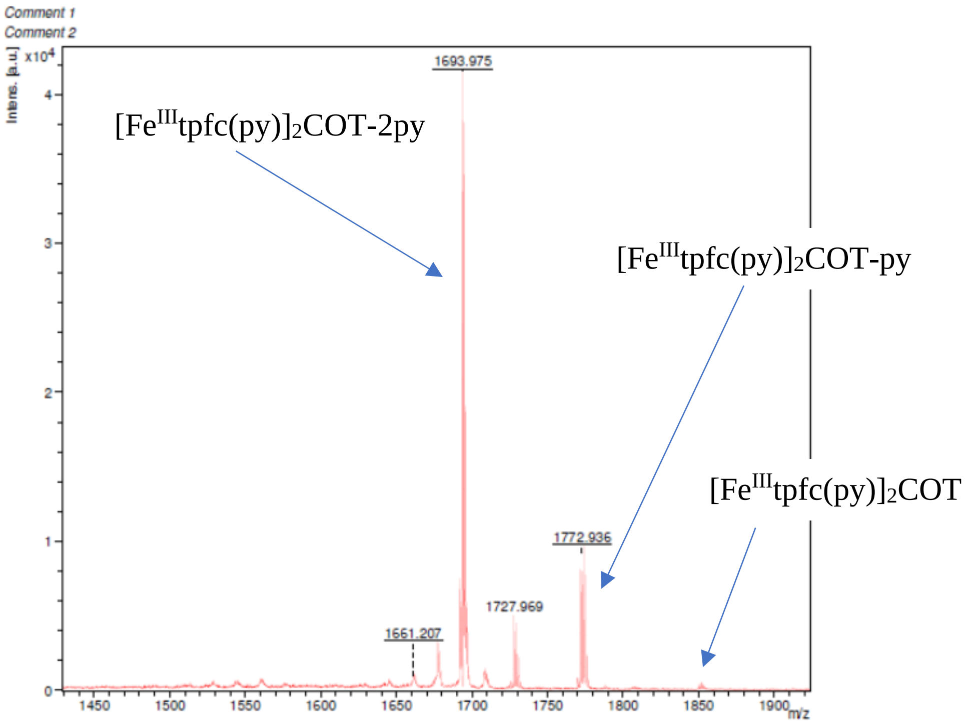

Acquisition Parameter

Date of acquisition	2022-06-29T18:30:59.268+03:00
Acquisition method name	D:\Methods\flexControlMethods\Specification\RP_Imaging_200-1500_Da.par
Aquisition operation mode	Reflector
Voltage polarity	POS
Number of shots	200
Name of spectrum used for calibration	
Calibration reference list used	PeptideLipids mono

Instrument Info

User	tof-user
Instrument	FLEX-PC
Instrument type	autoflexTOF/TOF

Figure S8: MS$^+$ (TOF) of [Fe^{III}tpfc(py)]$_2$COT.

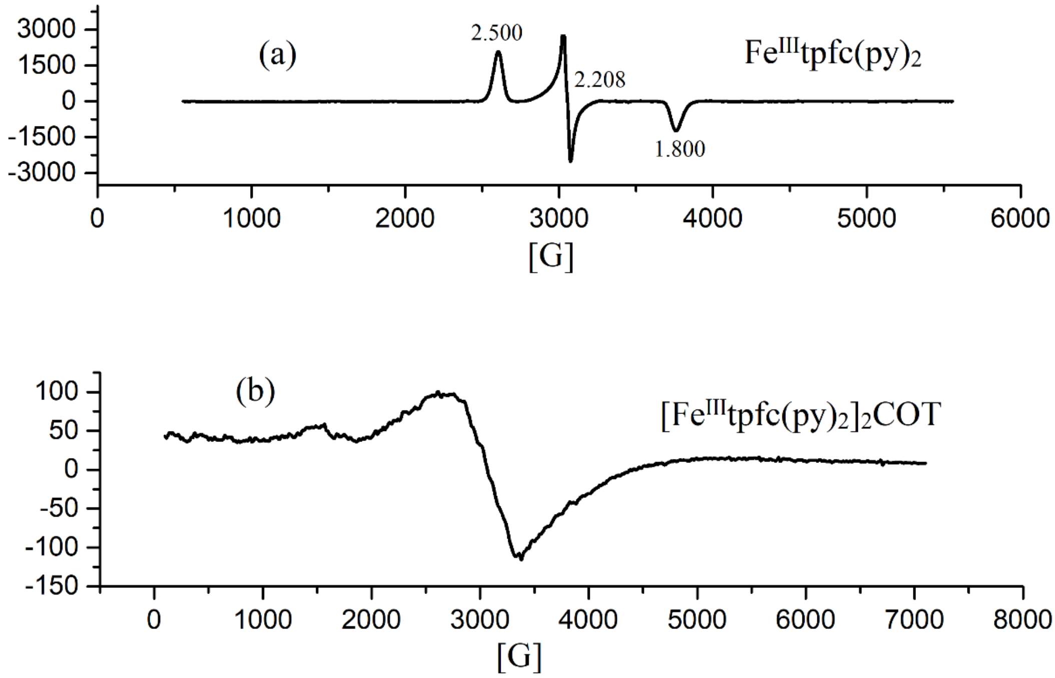

Figure S9: The EPR (1:1:1 benzene:$CHCl_3$:pyridine; 20K) of (a) Fe^{III}tpfc(py)$_2$ (g_x = 1.800, g_y = 2.08, g_z = 2.500) and (b) [Fe^{III}tpfc(py)$_2$]$_2$COT (g_1 = 1.7, g_2 = 2.0, g_3 = 2.24 with line width 664 G).

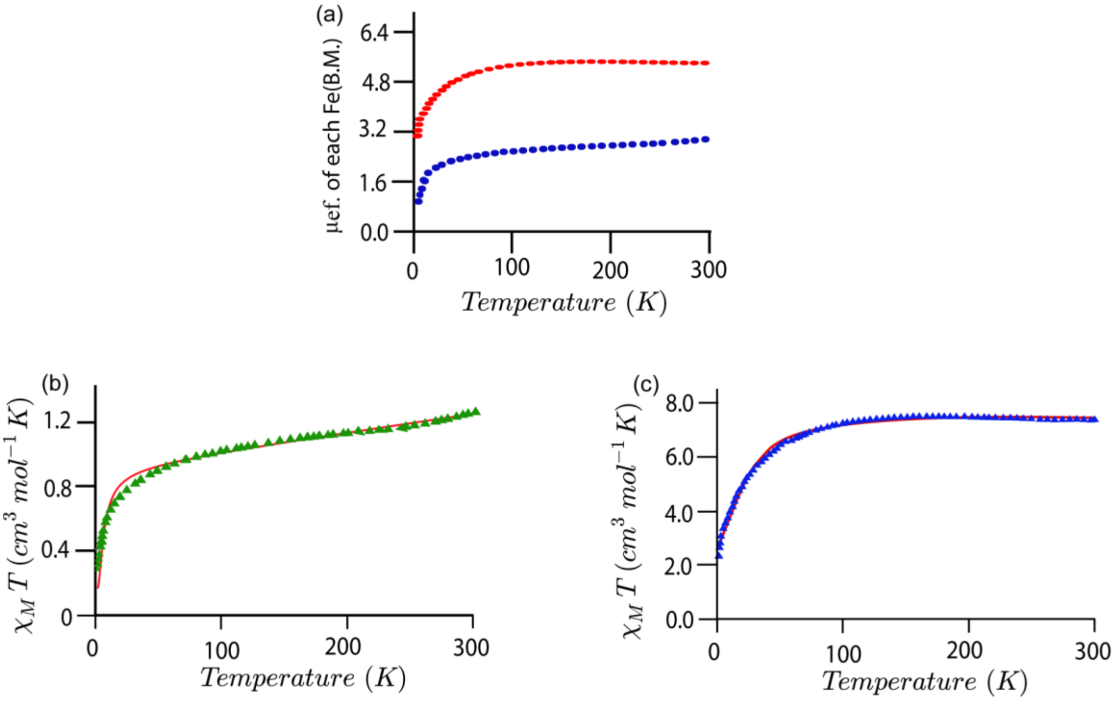

Figure S10: Variable temperature magnetic susceptibility measured on microcrystalline samples in the range of 2-300K by using SQUID magnetometry for: (a) $[Fe^{III}tpfc(py)]_2COT$ (red circles) and $[Fe^{III}tpfc(py)_2]_2COT$ (blue circles); (b) $[Fe^{III}tpfc(py)_2]_2COT$ (green triangles: observed, red line: simulated); and (c) $[Fe^{III}tpfc(py)]_2COT$ (blue triangles: observed, red line: simulated).

The magnetic susceptibility data were fitted using the software PHI.[2,3]

The calculated $\mu_{effective}$ for $[Fe^{III}tpfc(py)_2]_2COT$ is 1.08 BM at 2K (theoretical $\mu_{effective}$ per Fe atom = 1.8 BM) and the simulated metal-metal exchange interactions is J = -4 cm^{-1} at 2K. The calculated $\mu_{effective}$ for $[Fe^{III}tpfc(py)]_2COT$ is 3.03 BM at 2K (theoretical $\mu_{effective}$ per Fe atom = 3.8 BM) and the simulated metal-metal exchange interactions is J = -10 cm^{-1} at 2K.

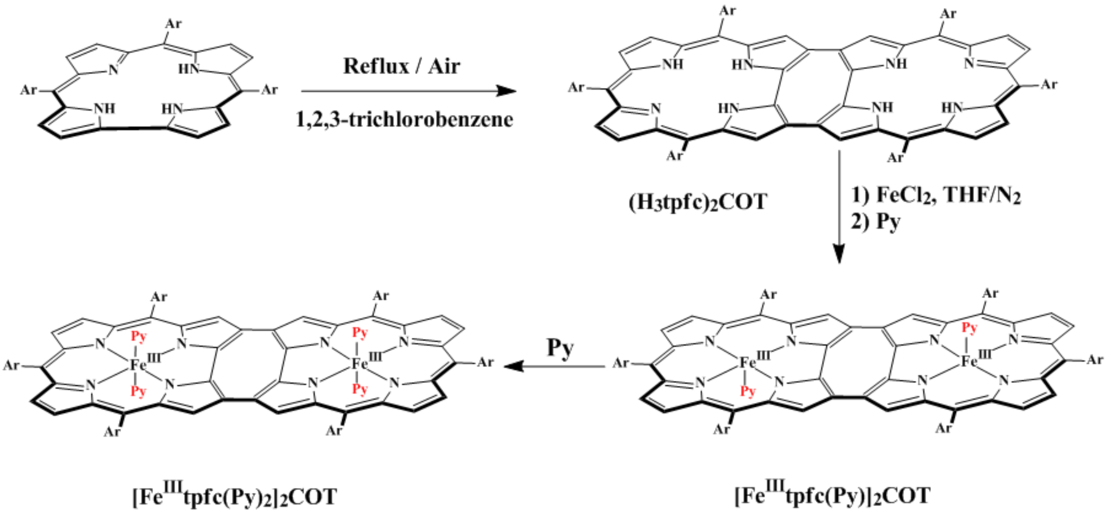

Scheme S1: Synthetic pathways for [Fe^{III}tpfc(py)]$_2$COT and [Fe^{III}tpfc(py)$_2$]$_2$COT.

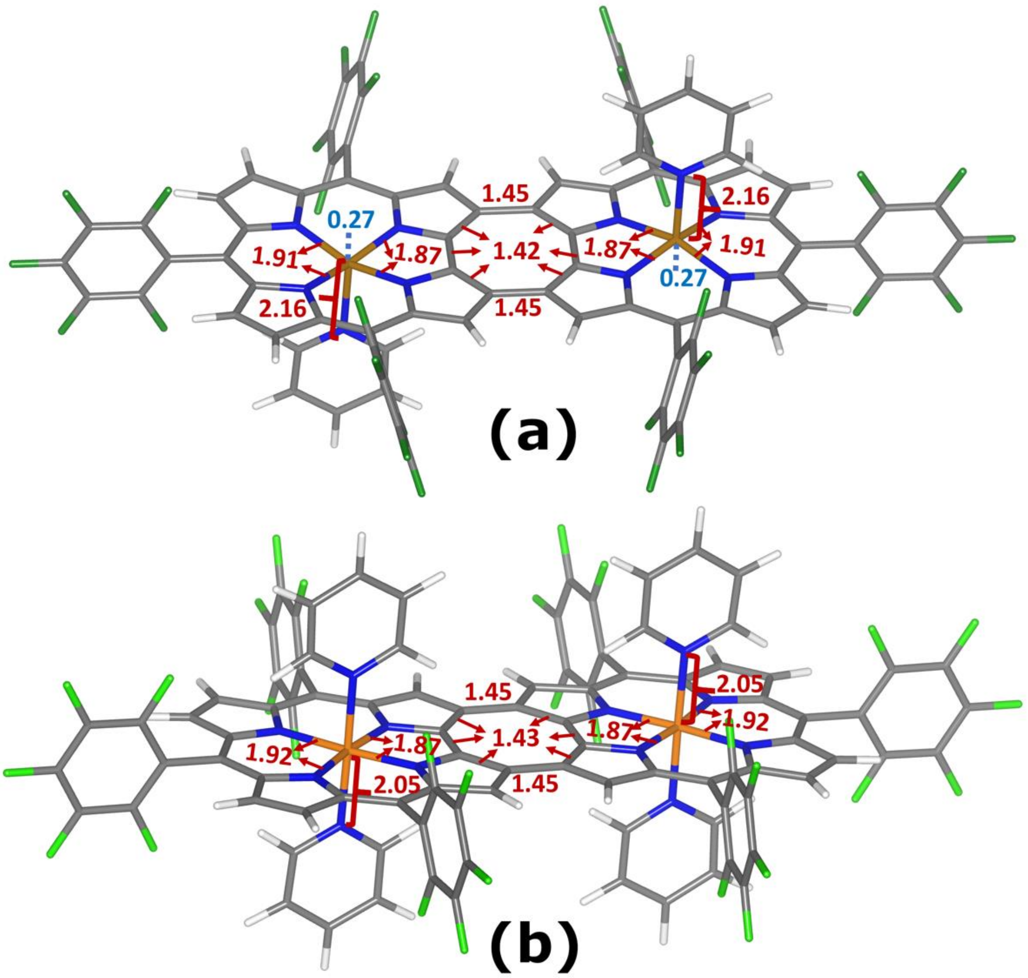

Figure S11: Optimized geometries of (a) the [Fe^{III}tpfc(py)]$_2$COT complex, with the Fe(III) being 0.27 Å above the macrocyclic ring, forming a domed-shaped structure,

and (b) the planar $[Fe^{III}tpfc(py)_2]_2COT$ complex. Important bond distances are given in angstrom.

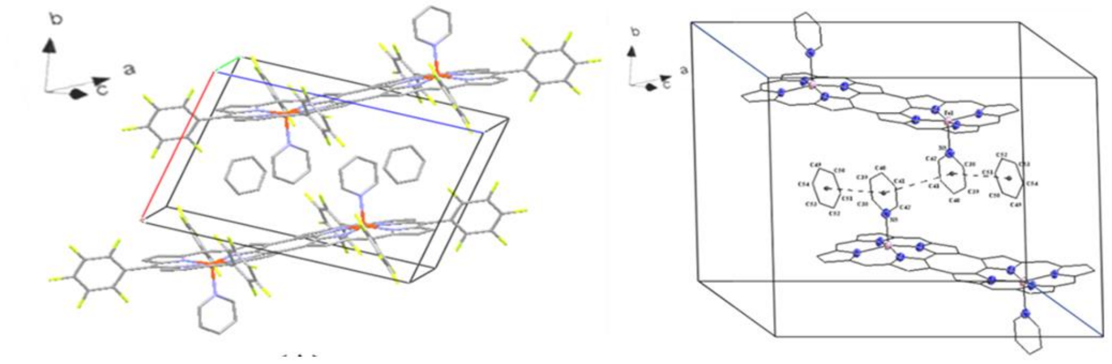

Figure S12: Left: Packing diagram of $[Fe^{III}tpfc(py)]_2COT$. Right: π-π stacking interaction between axial pyridine and benzene solvent molecule.

Table S1: Structural parameters for the bis-corrole dimers and monomeric iron corroles analogues.

Compound		M-N$_c$ [Å]^a	M-N$_{ax}$ [Å]^a	$\Delta^{Fe}{}_{23}$[Å]^b	Δ_{23}[Å]^c	$C_\beta{}^d$	$C_m{}^e$	reference
$[Fe^{III}tpfc(py)]_2COT$		1.892(4)	2.148(6)	0.39	0.07	0.1	0.01	This work
$[Fe^{III}tpfc(py)_2]_2COT$		1.8635(5)	2.019(7)	0.0014	0.04	0.05	0.04	This work
$Fe^{III}OEC(py)$		1.893(2)	2.188(2)	0.42	0.21	-	-	4
$Fe^{III}tpfc(py)_2$	Mol-I	1.887(5)	2.021(5), 2.037(5)	0.07	0.06	0.05	0.01	5
	Mol-II	1.883(5)	2.022(5), 2.042(5)	0.05	0.05	0.04	0.09	

^{a} Bond distances. ^{b}Displacement of Fe from the least-squares plane of $C_{19}N_4$ corrole core. ^{c}Average displacement of atoms from the least-squares plane of $C_{19}N_4$ corrole core. ^{d}Average displacement of β carbon atoms from the least-squares plane of $C_{19}N_4$ corrole core. .^{e}Average displacement of *meso*-carbon atoms from the least-squares plane of the $C_{19}N_4$ corrole core.

Table S2: comparison of the C-C bond lengths (marked as a-d according to the Figure) within the COT moiety as determined by X-ray crystallography.

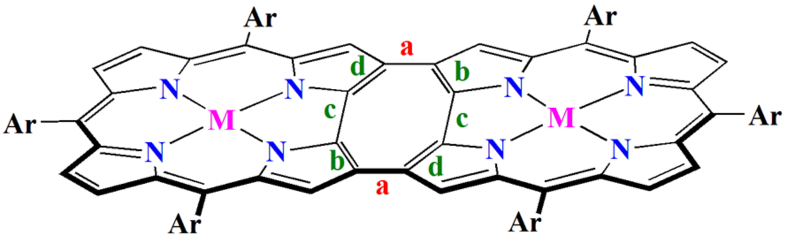

Compound	a	B	C	D
[(Fe-tpfc)$_2$]COT(py)$_2$	1.451	1.425	1.418	1.415
[(Fe-tpfc)$_2$]COT(py)$_4$	1.422	1.416	1.414	1.416
[(Ga-tpfc)$_2$]COT(py)$_2$	1.453	1.432	1.432	1.432
[(Ga-tpfc)$_2$]COT(py)$_4$	1.445	1.423	1.423	1.422

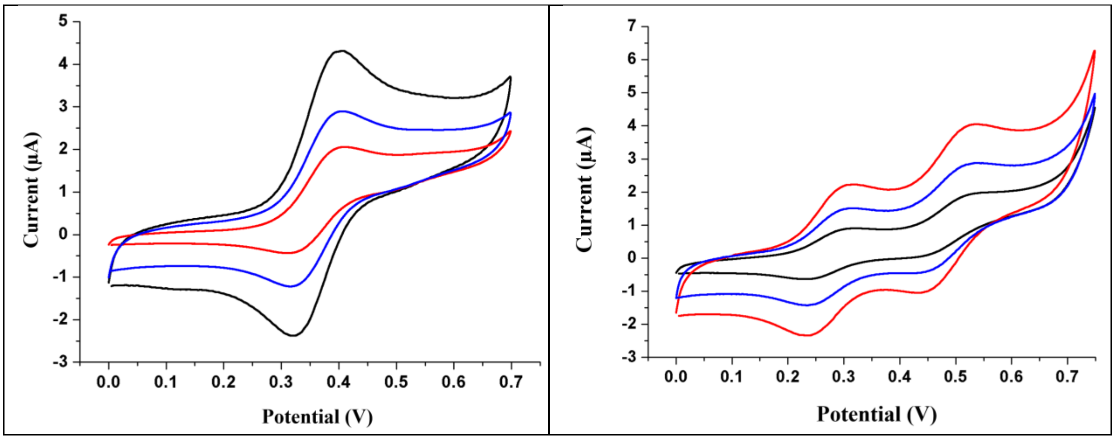

Figure S13: Cyclic voltammograms of Fe^{III}tpfc(py) (left) and [Fe^{III}tpfc(py)]$_2$COT (right) at 100, 250 and 500 mV/sec. Conditions: 0.50 mM complex, 0.1 M TBAP, Argon saturated DMF. Working electrode – glassy carbon, counter electrode – Pt wire, reference electrode – Ag/$AgNO_3$. $E_{1/2}$ (Ferrocene) = 0.075 V.

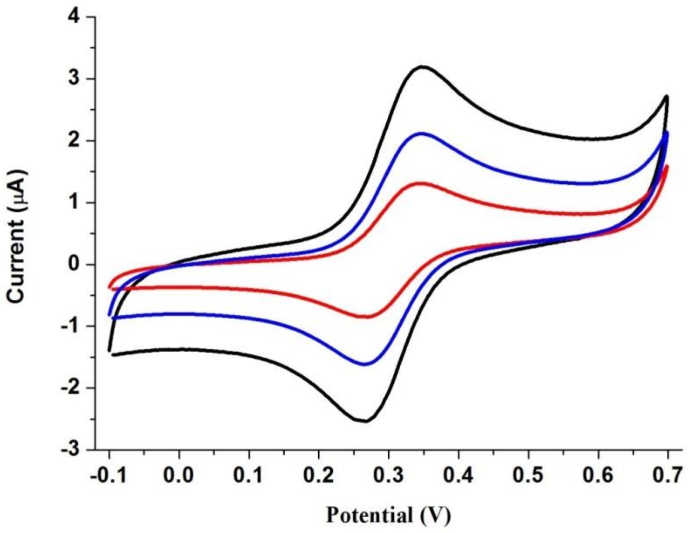

Figure S14: Cyclic voltammogram of 0.50 mM Ga^{III}tpfc(py) at 100, 250 and 500 mV/sec measured in Argon saturated DMF, 0.1 M TBAP. Working electrode – glassy carbon, counter electrode – Pt wire, reference electrode – Ag/$AgNO_3$. $E_{1/2}$ (Ferrocene) = 0.09 V.

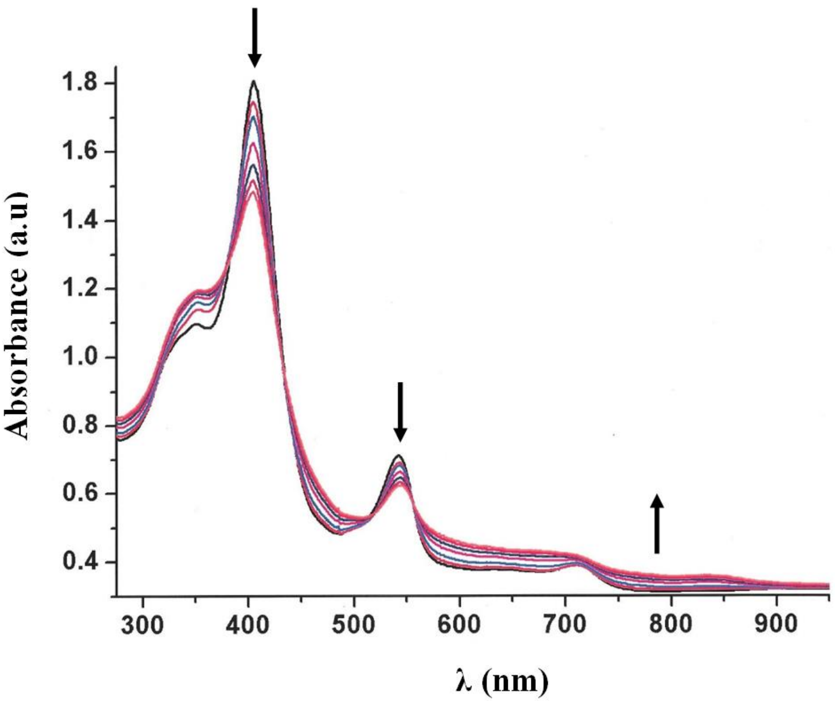

Figure S15: UV-Vis spectral changes of 0.25 mM Fe^{III}tpfc(py) during controlled potential oxidation at +0.6 V. 0.2 M TBAP, Argon saturated DMF. Working electrode – Pt gauze, counter electrode – Pt wire, reference electrode – Ag/$AgNO_3$.

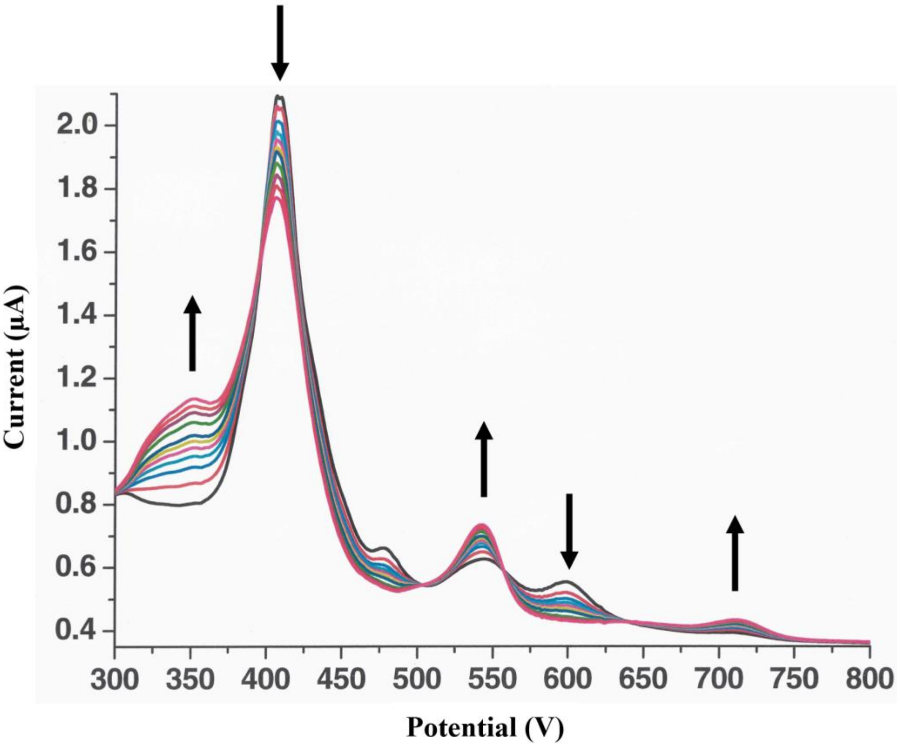

Figure S16: Re-oxidation of $[Fe^{II}tpfc(py)]^{-}$ during controlled potential oxidation at -0.2 V. 0.2 M TBAP, Argon saturated DMF. Working electrode – Pt gauze, counter electrode – Pt wire, reference electrode – $Ag/AgNO_3$.

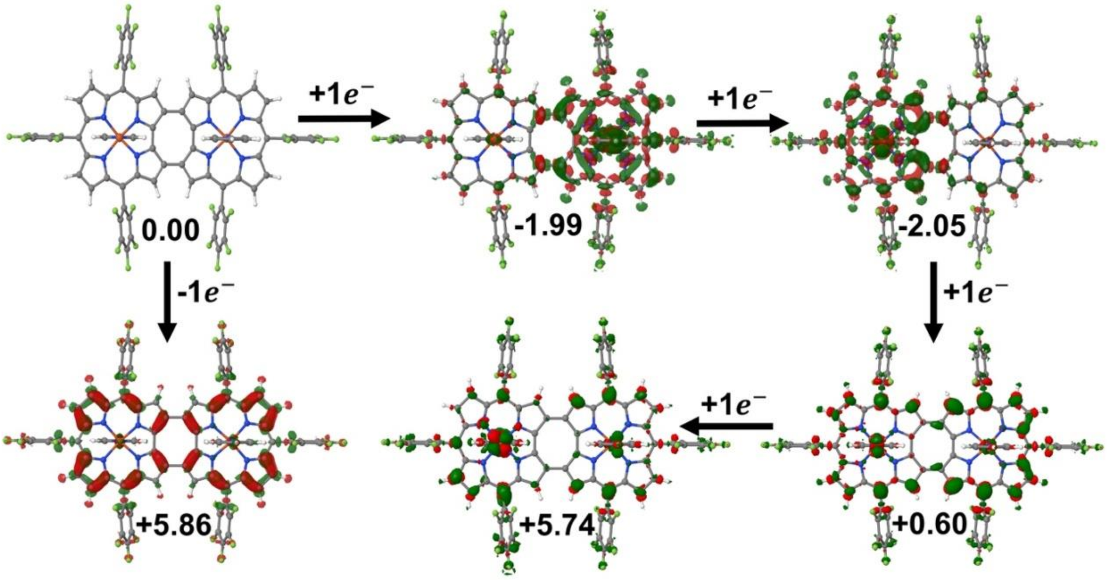

Figure S17: Differences in self-consistent charge-density, calculated for the COT-fused Fe (III) corrole dimer, relative to the neutral state (except for the two-, three- and four-electron states, which are relative to the one-, two- and three-electron state, respectively). An iso surface value of 0.001 electrons/bohr^3 was used. Electron addition and removal energies are in eV.

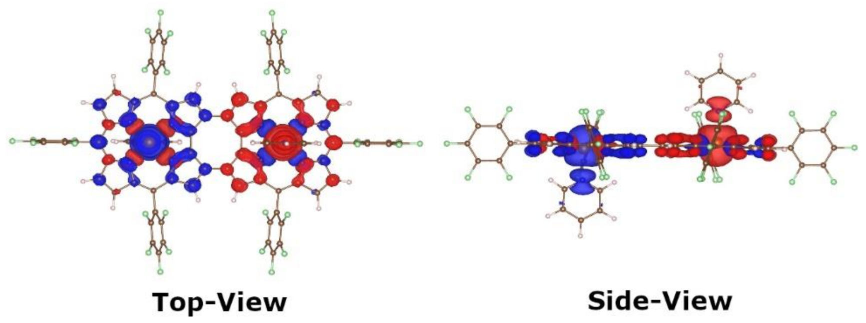

Figure S18: Spin-density distribution of the antiferromagnetic configuration.

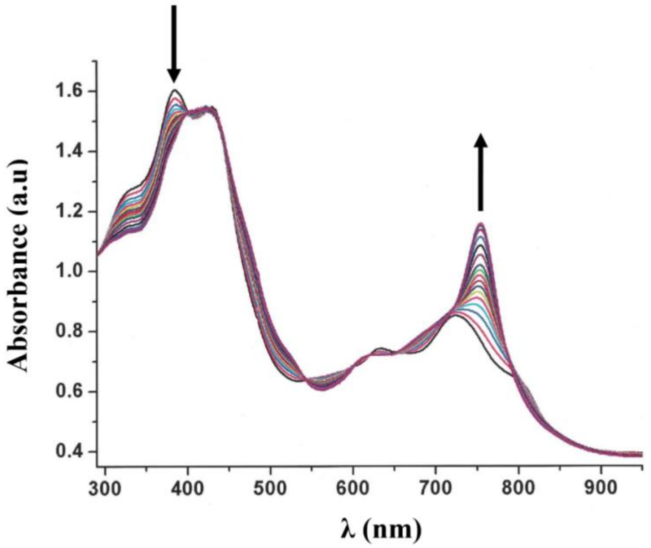

Figure S19: Spectroelectrochemistry of $[Fe^{III}tpfc(py)]_2COT$. 0.25 mM, 0.2 M TBAP, Argon saturated DMF, -1.5 V. Working electrode – Pt gauze, counter electrode – Pt wire, reference electrode – $Ag/AgNO_3$.

References:

1. Kahn, O. Molecular magnetism. VCH-Verlag, Weinheim, New York 1993. ISBN 3-527-89566-3, pp. 2-4.
2. Chilton, N. F.; Anderson, R. P.; Turner, L. D.; Soncini, A.; Murray, K. S. PHI: A powerful new program for the analysis of anisotropic monomeric and exchange-coupled polynuclear *d*- and *f*-block complexes. *J. Comput. Chem.* **2013**, *34*, 1164–1175.
3. Sil, D.; Dey, S.; Kumar, A.; Bhowmik, S.; Rath. S. P. Oxidation triggers extensive conjugation and unusual stabilization of two di-heme dication diradical intermediates: role of bridging group for electronic communication. *Chem. Sci.* **2016**, *7*, 1212–1223.
4. Vogel, E.; Will, S.; Talling, A. S.; Neumann, L.; Lex, J.; Bill, E.; Trautwein, A. X.; Wieghardt. K. Metallocorroles with Formally Tetravalent Iron. *Angew. Chem., Int. Ed. Engl*. **1994**, *33*, 731-735.

5. Simkhovich, L.; Mahammed, A.; Goldberg, I.; Gross, Z. Synthesis and Characterization of Germanium, Tin, Phosphorus, Iron, and Rhodium Complexes of Tris(pentafluorophenyl)corrole, and the Utilization of the Iron and Rhodium Corroles as Cyclopropanation Catalysts. *Chemistry - A European Journal* **2001**, *7* (5), 1041–1055.
